# Nanoparticles for Magnetic Heating: When Two (or More) Is Better Than One

**DOI:** 10.3390/ma14216416

**Published:** 2021-10-26

**Authors:** Jesus G. Ovejero, Federico Spizzo, M. Puerto Morales, Lucia Del Bianco

**Affiliations:** 1 Departamento de Energía, Medio Ambiente y Salud, Instituto de Ciencia de Materiales de Madrid, CSIC, Cantoblanco, E-28049 Madrid, Spain; jesus.g.ovejero@csic.es (J.G.O.); puerto@icmm.csic.es (M.P.M.); 2 Servicio de Dosimetría y Radioprotección, Hospital General Universitario Gregorio Marañón, E-28007 Madrid, Spain; 3 Dipartimento di Fisica e Scienze della Terra, Università di Ferrara, I-44122 Ferrara, Italy; federico.spizzo@unife.it

**Keywords:** magnetic hyperthermia, magnetic nanoparticles, magnetic aggregates, magnetic interactions, core/shell nanoparticles, multicore nanoparticles, hybrid systems, mixed nanoparticle systems, chemical synthesis, magnetic heating

## Abstract

The increasing use of magnetic nanoparticles as heating agents in biomedicine is driven by their proven utility in hyperthermia therapeutic treatments and heat-triggered drug delivery methods. The growing demand of efficient and versatile nanoheaters has prompted the creation of novel types of magnetic nanoparticle systems exploiting the magnetic interaction (exchange or dipolar in nature) between two or more constituent magnetic elements (magnetic phases, primary nanoparticles) to enhance and tune the heating power. This process occurred in parallel with the progress in the methods for the chemical synthesis of nanostructures and in the comprehension of magnetic phenomena at the nanoscale. Therefore, complex magnetic architectures have been realized that we classify as: (a) core/shell nanoparticles; (b) multicore nanoparticles; (c) linear aggregates; (d) hybrid systems; (e) mixed nanoparticle systems. After a general introduction to the magnetic heating phenomenology, we illustrate the different classes of nanoparticle systems and the strategic novelty they represent. We review some of the research works that have significantly contributed to clarify the relationship between the compositional and structural properties, as determined by the synthetic process, the magnetic properties and the heating mechanism.

## 1. Introduction

The amazing progress made in the last decade in the production of magnetic nanoparticles (NPs) for biomedical applications has been possible thanks to the close operational connection between chemistry, physics and biology. Taking advantage of the unique properties of the magnetic materials at the nanoscale it is possible to prepare colloids that can be remotely manipulated/stimulated/monitored through a magnetic stimulus [1].

Among the possible uses of magnetic NPs in nanomedicine, those based on their ability to produce heat under an alternating magnetic field (AMF) have been the subject of strong research interest for many years. Magnetic NPs can be exploited as heating agents in oncological treatments by taking advantage of the local heat generated in hyperthermia therapies or using this heat to trigger other thermosensitive therapies. The heat produced by NPs delivered at the tumor site can kill the cancer cells [2,3,4,5,6,7,8,9] or inhibit their self-renewal capacity [10]. Magnetic hyperthermia can enhance the effects of radiotherapy on cancer cells [11,12,13] and activate the immune system to fight metastatic tumors [14]. In a different strategy, magnetic NPs can be incorporated into a biocompatible matrix together with drug molecules (bound to the NPs or loaded separately) and magnetic heating can be used to induce the controlled degradation of the matrix and the targeted release of the drug, maximizing its effect and monitoring the treatment [6,15,16,17,18,19,20,21,22,23]. Comprehensive review articles can be found in the literature, well illustrating the latest advances and future prospects in biomedical applications of magnetic heating through NPs [24,25,26,27,28,29].

Novel types of magnetic NPs are continuously being designed with the ultimate goal of improving heating performance and biocompatibility. In this process, the knowledge acquired over time on the magnetism of nanosized structures and on the physics of the heat generation mechanism directs the efforts for the chemical synthesis of NPs with controlled magnetic properties and tuned heating capacity. On the other hand, the preparation of NPs with innovative structural and compositional characteristics offers the possibility to reveal or better elucidate peculiar magnetic behaviors and thus to expand the fundamental comprehension of magnetic phenomena at the nanoscale.

The development of the synthetic methods of colloidal magnetic NPs has achieved a fine control on their size, on the degree of crystallinity and also on their shape [30], which are key parameters that determine the magnetic properties, particularly the anisotropy energy barrier associated with the reversal of the magnetic moment [31,32]. A second important aspect on the development of functional magnetic materials is the effect of the surface chemistry on the colloidal properties and NP reactivity. In most preparations, the NPs are coated with a non-magnetic layer (organic, inorganic, ceramic or metallic) created on purpose or grown as a natural consequence of the synthetic process. The coating (i) confers biocompatibility to the NPs, (ii) determines their hydrophobic or hydrophilic character and colloidal stability and (iii) introduces intermediate moieties for the attachment of drugs or other biofunctional molecules (enzymes, proteins, antibodies) [33]. From the magnetic point of view, the presence of a non-magnetic coating rules the magnetic interactions, preventing the exchange coupling—which only occurs between NPs in close contact—and modulating the strength of dipolar interactions. Furthermore, coatings could even modify the magnetic properties of small magnetic cores if they bind covalently to the surface atoms perturbing the electronic state [34].

**Figure 1:**
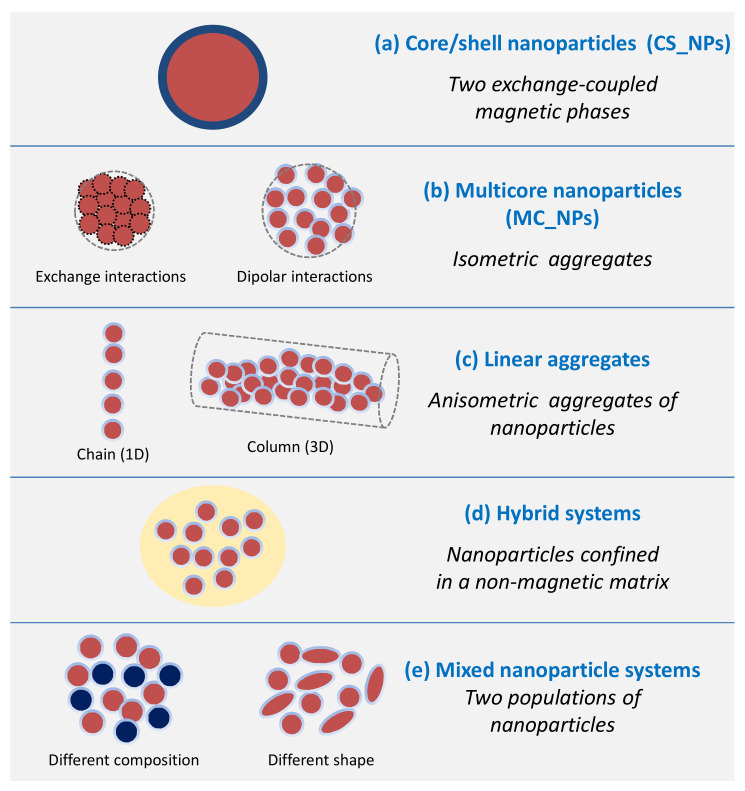
Scheme showing the different classes of magnetic NP systems whose magnetic heating performance is determined by the synergy between two or more constituent magnetic elements.

Over the last decade, the growing interest for magnetic heating agents has led to the development of new synthesis protocols for the creation of NPs consisting of two magnetic phases, forming a core/shell structure, or of systems comprising several distinguishable magnetic elements arranged in complex architectures. In this review, we present some of the most relevant examples of chemically synthesized systems that exploit the magnetic coupling between two or more constituent magnetic elements (magnetic phases, primary NPs) to enhance and tune the heating efficiency. We will focus on those works that have significantly contributed to clarify the connection between the compositional and structural properties, analyzing in detail the synthetic process, the magnetic properties and the heating mechanism. Since these elements are known to be strictly intertwined, elucidating their relationship is never trivial. In fact, each new material has its own peculiarities and the elements of knowledge acquired for a system cannot be applied directly and uncritically to another, but an in-depth study is needed. For this reason, the aim of the review is to analyze the most common strategies followed to optimize the heating performance of interacting magnetic systems. Thus, after a brief presentation of the fundamental principles underlying the magnetic heating mechanism, highlighting the principal structural and magnetic parameters involved in it, we will address the following classes of magnetic systems (Figure 1):

(a) Core/shell nanoparticles (CS_NPs): NPs with a core/shell structure made of two different magnetic phases, typically iron/iron-oxide and hard/soft ferromagnets. The magnetic properties are ruled by the magnetic exchange coupling between the two phases;
(b) Multicore nanoparticles (MC_NPs): Nanosized isometric (i.e., sphere-like) structures comprising more cores, i.e., primary NPs, of the same magnetic phase. The cores are structurally connected or held together by chemical bonds and are subjected to exchange or dipolar magnetic interactions;
(c) Linear aggregates: Anisometric assemblies of dipolar interacting NPs (chains and columns, namely 1-dimensional and 3-dimensional structures, respectively);
(d) Hybrid systems: Hybrid magnetic materials, i.e., consisting of magnetic NPs incorporated in a matrix with different chemical nature, such as lipid structures, polymers or silica. Attention will be focused on systems structured in such a way that the NPs are confined in a delimited spatial region and thus inevitably form magnetic aggregates;
(e) Mixed NP systems: Assemblies obtained by mixing together two populations of NPs differing in composition and/or shape, which implies a different magnetic anisotropy and possibly different colloidal properties.

Most of the systems described in this review are made up, in whole or in part, of iron oxide. The term ‘magnetic iron oxide’, which is widespread in literature on magnetic NPs, is generally used to indicate the ferrimagnetic iron oxide phases, i.e., magnetite (Fe_3_O_4_) and maghemite (γ-Fe_2_O_3_). These phases present a good degree of biocompatibility and iron oxide in the form of NPs is one of the few inorganic nanosystems approved by the U.S. Food and Drug Administration (FDA) for use in human patients. As maghemite can result from the oxidation of magnetite, iron oxide NPs are often reported to consist of a mix of these two phases [35,36]. On the other hand, the two phases have similar inverse spinel structures, which are very difficult to be experimentally distinguished at the nanoscale.

This review does not include all the reported cases of magnetic NPs which show the unwanted and uncontrollable tendency to aggregate during the synthesis or during the magnetic heating process under the action of the AMF. We exclusively focus on designed NP systems for magnetic heating applications, prepared by chemical methods, which possess as strength point the synergistic union of different magnetic components.

## 2. Basis of the Magnetic Heating Phenomenon

This section summarizes the basic physics concepts on the phenomenon of magnetic heating generated by NPs, in order to highlight the main structural and magnetic parameters involved in it. To deepen the subject, we refer the reader to the article by Carrey et al. [37] and to the review by Perigo et al. [38]. In particular, the article by Carrey et al. is a rigorous presentation of the theory behind the magnetic heating effect, which has also the merit to clearly point out the improper separation, made in several experimental articles, of the mechanisms responsible for the heating between ‘hysteresis losses’, produced by NPs in the ferromagnetic regime, and ‘relaxation losses’ produced by NPs in the superparamagnetic regime. This separation is misleading since the heat released by an assembly of magnetic NPs under an AMF, per unit volume and during one cycle, is equal to the area A of the resulting hysteresis loop (magnetization vs. AMF field amplitude). Therefore, the magnetic energy losses are always hysteresis losses. The Specific Absorption Rate (SAR) parameter quantifies the efficiency of the NPs to transform magnetic energy into heat and corresponds to the product between A and the frequency f_m_ of the applied AMF. According to this definition, SAR is expressed in W/m^3^ units (SI system). Usually, the SAR quantity is given in W/kg units, which is obtained by dividing by the mass density ρ of the NPs, namely

(1) $$\mathrm{SAR}={\mathrm{Af}}_{m}/\rho \;\;\;[W/\mathrm{kg}]$$

It is well known that, below a critical size, a magnetic NP becomes single domain, in order to minimize the magnetostatic energy, and its magnetic moment lies along one of the magnetic anisotropy axes (i.e., the easy magnetization directions). In the case of uniaxial anisotropy, the moment has only two stable orientations separated by an energy barrier KV, where K is the magnetic anisotropy coefficient and V is the NP volume. The action of an externally applied field H on the NP is described by the Stoner-Wohlfarth model, which essentially provides the magnetization M vs. H at different values of the angle θ between the anisotropy axis and the applied field [32].

Two limiting cases can be distinguished. (i) When the field is parallel to the anisotropy axis (θ = 0), a perfectly squared hysteresis loop is obtained with maximal area given by

(2) $$A={4\mu }_{0}M_{S}H_{K}=8K$$

where M_S_ is the NP saturation magnetization (in this case, M_S_ coincides with the remanent magnetization M_r_) and µ_0_H_K_ is the anisotropy field, which corresponds to 2 K/M_S_ (in this case, anisotropy field, switching field and coercivity H_C_ coincide). (ii) When the field is perpendicular to the anisotropy axis (θ = π/2) no magnetic hysteresis is observed.

In the case of an assembly of non-interacting randomly oriented identical NPs, the hysteresis loop features a remanent magnetization M_r_ = 0.5 M_S_ and a coercivity H_C_ = 0.48 µ_0_H_K_. Accordingly, the loop area is reduced and it can be approximately estimated as

(3) $$A\approx {4\mu }_{0}M_{r}H_{C}={2\mu }_{0}M_{S}H_{C}=1.92K$$

The Stoner–Wohlfarth model considers the temperature T = 0 K, i.e., does not take into account thermal effects on the magnetization process. However, temperature activates magnetic relaxation processes, i.e., the thermal energy can assist the magnetic field in promoting the reversal of the NP moment. Therefore, the coercivity decreases on increasing temperature. Moreover, when the anisotropy energy barrier is comparable to the thermal energy or lower, the moment overcomes the energy barrier without the need of an applied field and is free of thermally fluctuating between the two energy minima corresponding to the stable orientations, similarly to the atomic spins of a paramagnetic material. This phenomenon is known as superparamagnetic relaxation. An assembly of superparamagnetic NPs can be brought to magnetic saturation by an external field, but it does not exhibit magnetic hysteresis, i.e M_r_ and H_C_ are null.

Magnetic relaxation effects can be dealt with in the framework of the Néel relaxation theory and hence considering the existence of a relaxation time for the moment reversal. Under the assumption that the atomic spins of the NP rotate coherently in the reversal process (macrospin approximation), the Néel expression for the relaxation time of the NP moment is:

(4) $$\tau_{N}=\tau_{0}{\exp(\mathrm{KV}/k}_{B}T)$$

where k_B_ is the Boltzmann constant, hence k_B_T is the thermal energy, and τ_0_ is the flipping time. The latter inversely depends on the gyromagnetic ratio γ_0_, usually given in angular frequency [39], and it is generally assumed equal to 10^−9^ s.

The observed magnetic behavior of the NP depends on the value of τ_N_ with respect to the measuring time t_m_ characteristic of the used investigating technique (f_m_ = 1/t_m_ is the measuring frequency). The NP is in the superparamagnetic regime for τ_N_ < t_m_ (or for f_m_τ_N_ < 1) and in the blocked ferromagnetic regime for τ_N_ > t_m_ (f_m_τ_N_ > 1). Conventionally, the transition between the two regimes occurs at τ_N_ = t_m_ (i.e., f_m_τ_N_ = 1). It follows that the temperature T_B_ (blocking temperature) that marks the passage between the blocked regime and the superparamagnetic one is expressed by the relation (in which f_0_ = 1/τ_0_):

(5) $$T_{B}={\mathrm{KV}/k}_{B}{\ln(t}_{m}f_{0})$$

Therefore, T_B_ shifts to higher values with reducing t_m_, i.e., with increasing f_m_. For DC measurements by SQUID (Superconducting Quantum Interference Device) magnetometer, a value t_m_ = 100 s is usually considered (i.e., measuring frequency f_m_ = 0.01 Hz).

Accordingly, for a fixed temperature T, the critical volume above which a NP is blocked and below which it is superparamagnetic is:

(6) $$V_{P}=\ln(t_{m}f_{0})k_{B}T/K$$

For instance, from the above equation, at t_m_ = 100 s and T = 300 K, the critical diameter for a spherical magnetite NP is ~26 nm, setting K equal to the magnetocrystalline anisotropy of the bulk phase (1.1 × 10^5^ erg/cm^3^).

Regarding the magnetic hysteretic properties of an assembly of NPs subjected to an AMF with amplitude H_max_ and frequency f_m_, two principal scenarios can be distinguished, depending on the value of the parameter ξ = µ_0_M_S_VH_max_/k_B_T.

The first is described by the so-called Linear Response Theory (LRT), which assumes that the magnetization M is linear with the magnetic field [37,40]. This assumption substantially coincides with the condition ξ < 1, which is therefore a fundamental requirement for the applicability of LRT. Hence, for fixed M_S_ and V, the LRT theory ceases to be valid at sufficiently high H_max_ values. Moreover, it is possible to show that LRT can be more adequately applied to strongly anisotropic NPs [37].

The hysteresis loop area A for randomly oriented NPs, predicted by LRT, is related to the imaginary component of the magnetic susceptibility χ″ through this relation

(7) $$A={\pi \mu }_{0}H_{\max}^{2}\;\chi^{\prime \prime }=\frac{{\pi \mu }_{0}^{2}H_{\max}^{2}M_{S}^{2}V}{{3k}_{B}T}\frac{f_{m}\tau_{N}}{1+({f_{m}\tau_{N})}^{2}}$$

It is worth noticing that the AC magnetic susceptibility, as obtained from the Casimir–Du Pré model [41], depends on ϖτ_N_ where ϖ corresponds to 2πf_m_, actually. However, as observed by Dormann et al. [39,42], if the gyromagnetic ratio γ_0_ is given in angular frequency, ϖ has to be replaced by f_m_. Accordingly, for AC magnetic measurements, the measuring frequency, that is the reciprocal of the measuring time t_m_, coincides with the field frequency (for this we have indicated them both as f_m_) [39,42]. Therefore, a resonant phenomenon is essentially observed on varying f_m_. In fact, according to Equation (7), A is null for f_m_τ_N_ << 1 (full superparamagnetic regime) and for *f*_m_τ_N_ >> 1 (full ferromagnetic regime) and reaches a maximum for f_m_τ_N_ = 1 (transition between the two regimes), which is the resonant condition. Apart from a few exceptions [43,44,45,46], most studies on the heating properties of magnetic NPs refer to the LRT, even when the criterion ξ < 1 is not fulfilled. Indeed, if the condition ξ < 1 is not satisfied, a different scenario opens. Regarding the fully superparamagnetic NPs, also in this case they are useless for generating heat because of their null hysteresis. The loop area of single-domain NPs in the full ferromagnetic regime can be predicted by the Stoner–Wohlfarth model, eventually including also the thermal dependence of the coercivity, namely considering A(T) ~ 4µ_0_M_r_H_C_(T). This approach is valid under the assumption that the assembly is substantially saturated by H_max_, which is not always the case, actually. Highest area values are reached by adjusting H_max_ well above the anisotropy field H_K_. The loop area increases with increasing H_max_ up to the value at which magnetic saturation is attained. For a fixed H_max_, the SAR parameter increases with increasing f_m_, unlike what occurs in the LRT frame, in which the loop area and hence the SAR are maximized by setting f_m =_ 1/τ_N_.

In magnetic heating experiments, care should be taken to select H_max_ and f_m_ so that their product does not exceed 5 × 10^9^ A/ms, which is indicated as the criterion to avoid detrimental effects on living organs in medicine applications [47].

Above a critical size, which depends on M_S_ and K, the description of the NPs as canonical single magnetic domains, whose atomic spins reverse coherently, is no longer valid and therefore the Stoner-Wohlfarth model cannot be applied. In fact, closure magnetization configurations, i.e., vortex-type, and incoherent reversal modes may become energetically favored, resulting in a lower coercivity and hence narrower hysteresis loops [48,49,50,51]. Obviously, the same is true for particles with a multi-domain configuration [31,52].

It should be also remarked that neither the Néel relaxation theory nor the Stoner-Wohlfarth model consider the existence of interparticle magnetic interactions. This is exactly one of the main points that we will address in the following, namely how the magnetic heating response of an assembly of magnetic nanoheaters is influenced by magnetic interactions.

When the NPs are dispersed in a fluid, another magnetization mechanism may be active besides the internal rotation of the moments, namely the physical rotation of the NPs due to the torque action exerted by the magnetic field and under the influence of thermal effects [40,53,54]. The process is usually described within the Brownian relaxation theory, by introducing a relaxation time defined as

(8) $$\tau_{B}={3\eta \;V}_{H}/k_{B}T$$

where η is the viscosity of the solvent and V_H_ is the hydrodynamic volume of the NP [40].

As in the Néel relaxation, the magnetic behavior of a NP subjected to Brownian relaxation depends on the value of τ_B_ with respect to the measuring time t_m_. Hence, the relaxation mechanism that ultimately rules the magnetic reversal behavior of the NP is that with the shortest relaxation time, under the adopted experimental conditions. The hydrodynamic volume V_H_ is usually larger than the physical one and can be strongly altered by the tendency of the NPs to aggregate during the synthetic process, under the action of electrostatic or magnetic interactions. Moreover, the application of the AMF during the heating tests can result in the formation of chains and agglomerates of NPs [55,56,57,58], namely V_H_ can change in an unpredictable way. Hence, Brownian motion is a quite difficult phenomenon to evaluate and govern in practice.

Operatively, the heating capacity of a NP assembly can be assessed through a calorimetric method, namely by measuring the temperature increase during time of a fluid containing a certain amount of NPs, subjected to the AMF. The SAR parameter is calculated using the relation [59]

(9) $$\mathrm{SAR}=\frac{C}{m_{\mathrm{NPs}}}\cdot \frac{{\Delta T}_{}}{\Delta t}$$

where C is the heat capacity of the sample (taken equal to the heat capacity of the fluid, if that of the NPs is assumed negligible), m_NPs_ is the mass of the magnetic NPs and ΔT is the temperature increase during the time interval Δt. In the initial slope method, which is probably the most often used, ΔT/Δt is calculated as the slope of the linear curve fitting the initial portion of the heating curve.

The SAR parameter estimated by Equation (9) is usually given in W/g units or, in the case of ferrite NPs, in W/g_Fe_ units (i.e., watts per gram of iron). In literature, the same physical quantity expressed by SAR can be found indicated with different names: specific loss power (SLP), specific power loss (SPL), specific heat power (SHP), specific power absorption (SPA).

Another parameter, the intrinsic loss power (ILP), has been also proposed, given by

(10) $$\mathrm{ILP}=\frac{\mathrm{SAR}}{f_{m}H_{\max}^{2}}$$

ILP is defined on the assumption that SAR depends quadratically on H_max_ and linearly on f_m_, namely in the LRT context. Thus, normalizing SAR by these dependences should allow the heating efficiency at different experimental conditions of the applied field to be directly compared [60].

However, at present, the wide variety of customized instruments for magnetic heating tests and the lack of standardized protocols make it very difficult, if not impossible, to compare SAR values measured on different NP systems. In order to be comparable, SAR values estimated using a calorimetric method should not refer just to tests carried out at the same field frequency and amplitude, but also in similar thermodynamic conditions [59]. Moreover, it would be advisable to disperse the NPs at a similar concentration and in the same solvent.

## 3. Core/Shell Nanoparticles (CS_NPs)

The concept behind CS_NPs, consisting of two different magnetic phases, is to exploit the interface exchange coupling to tune the magnetic anisotropy and therefore the hysteretic properties (coercivity, remanent magnetization). In fact, this magnetic coupling can give rise to an additional source of anisotropy, i.e., exchange anisotropy, for the magnetically softer component, as first observed by Meiklejohn and Bean, decades ago, in ferromagnetic/antiferromagnetic Co/CoO CS_NPs [61]. Since then, the exchange coupling mechanism has been studied in a number of different systems (NPs, films and patterned structures) consisting of at least two magnetic phases with different intrinsic anisotropy and, in many cases, also with different magnetic nature (ferromagnetic, antiferromagnetic, ferrimagnetic). The exchange anisotropy is also responsible for the exchange bias effect [61,62,63,64,65], i.e., the horizontal shift of the hysteresis loop, particularly interesting for technological applications in spintronic devices [66].

In ferromagnetic/antiferromagnetic and ferromagnetic/ferrimagnetic CS_NPs, the exchange anisotropy produces an increase in coercivity, compared to that measured in the single-phase ferromagnetic cores [63,67,68,69]. Moreover, the exchange bias effect can appear when the assembly is cooled in a static magnetic field across a critical temperature, below which the anisotropy energy of the harder component (antiferromagnetic or ferrimagnetic, possibly showing a spin-glass-like behavior) is larger than the exchange interaction energy at the interface with the soft ferromagnetic phase. This is the case of iron/iron oxide NPs for example, whose exchange coupling has been strongly investigated [70,71,72,73,74,75,76]. CS_NPs of this type are particularly interesting because, thanks to the presence of the metallic iron core, the saturation magnetization is higher than that of iron oxide, but the oxide shell guarantees resistance to oxidation and biocompatibility.

In hard/soft ferromagnetic systems, the exchange anisotropy can produce a characteristic reversible demagnetizing curve (exchange-spring behavior) and, most remarkably, the coupling between the hard component, with high coercivity, and the soft component, with high saturation magnetization, results in a high value of the maximum energy product [77,78,79]. This phenomenon paved the way for the creation of a new generation of high-performance permanent magnets [80].

The chemical routes to produce CS_NPs includes co-precipitation in water [81,82], thermal decomposition in organic media, metal reduction in microemulsions [83], hydrothermal synthesis [84] and electrodeposition [85]. However, the most widely used and most efficient in terms of crystallinity, homogeneity and shape control is the two-step thermal decomposition method in organic media, named as seed-mediated method. On the first step, the core crystals are formed controlling carefully the size and surface orientation. The crystals formed are used in a second step to induce a heterogeneous nucleation of the shell phase to control the growth and avoid secondary nucleation [86,87,88,89]. Some examples of the materials explored are: Fe/CoFe_2_O_4_ [90]; CoFe_2_O_4_/ZnFe_2_O_4_ or ZnFe_2_O_4_/CoFe_2_O_4_ [91]; CoFe_2_O_4_/MnFe_2_O_4_ or MnFe_2_O_4_/CoFe_2_O_4_ [92]; Mn_x_Fe_3−x_O_4_/Fe_x_Mn_3−x_O_4_ [88]; Mn_3_O_4_/Fe_3_O_4_ or Fe_3_O_4_ /Mn_3_O_4_ [89]. A simpler alternative is the surface treatment of ferromagnetic metallic NPs to produce a thick and stable layer of antiferromagnetic or ferrimagnetic oxide, as is the case for Fe/Fe_3_O_4_ NPs [93,94,95,96]. Although simpler, this route offers a poor control on the shell thickness and low stability of phase composition due to oxygen migration towards the metallic core.

In this context, Zhang et al. were among the first to study iron/iron oxide CS_NPs for prospective biomedical applications (hyperthermia and magnetic resonance imaging) [97]. Their work was particularly focused on the surface engineering of the CS_NPs, in order to make them highly biocompatible. Iron NPs were synthesized by reduction of an iron salt by NaBH_4_ in a water-in-oil microemulsion solution of n-octane and water, in the presence of two surfactants, cetyl trimethyl ammonium bromide (CTAB) and n-butanol. The volume ratio between oil phase and water phase was increased to decrease the size of the iron NPs from 20 to 8 nm. The passivation procedure generated the core-shell structure of the NPs, with trimethylamine N-oxide ((CH_3_)_3_NO) that worked as a mild oxidant and flowing Ar for two days to improve the stability. Finally, phosphatidylcholine was assembled on the surface of the NPs to make them biocompatible. CS_NPs of ~20 nm in size, subjected to a AMF of 150 Oe at 250 kHz, could produce, in 60 s, a temperature increase higher than that obtained using single-phase iron oxide NPs.

Another interesting core/shell system with high M_S_ and good air stability was proposed by Meffre et al. [98]. It consisted of Fe(soft)/FeC(hard) NPs, prepared by first obtaining the iron metal cores by thermal decomposition of an iron organometallic compound [99] and then, adding Fe(CO)_5_ under H_2_ and heating at 120–150 °C. Final size of CS_NPs could be finely controlled between 12 and 15 nm by varying the average size of the initial iron(0) nanocrystals or the Fe(CO)_5_ concentration. To render the CS_NPs water soluble, the organic coating was exchanged with dimercaptosuccinic acid (DMSA). This method allowed the control of the amount of carbon diffused and therefore the tuning of the anisotropy of the CS_NPs. A SLP value of 415 W/g was measured in the best samples (AMF of 20 mT and frequency 96 kHz).

Tsopoe et al. carried out a comparative study on the exchange bias effect in antiferromagnetic/ferrimagnetic CS_NPs, with structure NiO/Fe_3_O_4_ and Fe_3_O_4_/NiO [100]. These structures were also synthesized in two steps: first the precipitation in water of the NiO or Fe_3_O_4_ core; then the precipitation of the other salt in the presence of the core and sodium acetate in ethylene glycol, in an autoclave at 180 °C for 10 h. CS_NPs between 30–35 nm showed colloidal stability thanks to the polyol rests at the surface. Both types showed higher SAR values in comparison to single-phase magnetite NPs, owing to the interface exchange coupling; the system with magnetite as shell exhibited higher exchange bias and SAR.

It is known that the structure of iron/iron oxide CS_NPs can deteriorate due to the interdiffusion of atoms between the core and the shell. This may even lead to a shrink of the core and to the formation of a hollow structure in the so-known Kirkendall effect [101]. The influence of this process on the heating efficiency was investigated by Nemati et al., studying Fe/γ-Fe_2_O_3_ CS_NPs obtained by thermal decomposition of Fe(CO)_5_ [102]. They found that with increasing the NP average size from 8 to 14 nm, the core/shell morphology was retained for a longer period of time and the heating efficiency improved. In hollow NPs, obtained by annealing the previous samples at 180 °C for one hour under oxygen, the heating efficiency decreased, rendering them less useful for magnetic hyperthermia application.

Famiani et al. also produced Fe/Fe_x_O_y_ CS_NPs with tunable sizes (12, 15, 18, and 20 nm) by thermal decomposition of Fe(CO)_5_ and used dopamine molecules to functionalize the iron oxide surface, replacing the native oleylamine groups through the catechol groups [103]. Larger sizes were obtained in this case by increasing the amounts of iron precursor and extending the injection time. The authors evaluated the retention of the stable magnetic α-Fe core upon exposure to air and after ligand exchange and its resulting effect on the magnetic hyperthermia.

Lee et al. exploited the hard/soft coupling mechanism to maximize the heating efficiency of magnetic ferrite NPs, different from magnetite and maghemite [104]. They studied CS_NPs made of a hard core of CoFe_2_O_4_ (9 nm in size) and a soft shell of MnFe_2_O_4_ (3 nm-thick). At T = 5 K, the coercivity was between the values of single-phase CoFe_2_O_4_ and MnFe_2_O_4_ NPs; the CS_NPs were superparamagnetic at room temperature. The SLP (tested in AMF of 37.3 kA/m and 500 kHz) was one order of magnitude larger than that of single-phase CoFe_2_O_4_ and MnFe_2_O_4_ NPs, with size 9 nm and 15 nm respectively. These CS_NPs were prepared by thermal decomposition in organic media, by the seed-mediated method. CoFe_2_O_4_ NP was used as a seed and synthesized by thermal decomposition of Co(acac) with 1,2-hexadecanediol in the presence of oleic acid and oleylamine [86]; MnFe_2_O_4_ was over-grown by thermal decomposition onto the surface of the seed NP, adding MnCl_2_ and Fe(acac)_3_ in the presence of oleic acid, oleylamine and trioctylamine, and heating at 365 °C/1 h. As-synthesized CS_NPs were transferred to the aqueous phase by modification of the surface using dimercaptosuccinic acid. Using the same synthetic method, the authors were able to prepare various core/shell combinations, including CoFe_2_O_4_/Fe_3_O_4_, MnFe_2_O_4_/CoFe_2_O_4_ and Fe_3_O_4_/CoFe_2_O_4_, demonstrating the possibility to tune the magnetic anisotropy of the CS_NPs and their SLP, which ranged between 1 and 4 kW/g. These remarkably high SLP values were obtained with the AMF indicated above, i.e., in testing conditions that did not fulfill the safety criterion for medicine applications [47], already mentioned in Section 2. It can easily be verified that the same consideration applies to many of the studies cited in this review, actually.

S. Liebana-Vinas et al. studied ferrite cores of soft MnFe_2_O_4_ or hard CoFe_2_O_4_ prepared by the same method described above, covered by a 2–3 nm Fe_3_O_4_ shell formed in a second step, with an overall size in the 10 nm range [105,106]. In both types of cores, the addition of the magnetite coating produced an improvement of the heating efficiency, but the effect was definitely more marked in the case of CoFe_2_O_4_, in which an increase of SLP by a factor of 24 was experienced [105]

Fabris et al. reported on the possibility to control the heat generation mechanism of colloids of Fe_3_O_4_/Zn_x_Co_1−x_Fe_2_O_4_ CS_NPs by changing the shell composition with a similar seed-mediated growth method [107]. In particular, they showed that the effective anisotropy of the whole core–shell structure could be tuned by the substitution of Co^2+^ by Zn^2+^ ions in the shell. Increasing the Zn concentration of the shell, from x = 0 to 1.00, decreased the magnetic anisotropy and, in turn, this effect provided a way to select the magnetic relaxation mechanism, Brown or Néel, which dominated the heating process.

Lavorato et al. synthesized by seed-mediated growth method Fe_3_O_4_/Co_x_Zn_1−x_Fe_2_O_4_ CS_NPs, whose heating properties could be optimized by modulating their shell composition and thickness and, hence, by finely controlling the interface exchange coupling and the resulting effective anisotropy [108]. They reported SLP up to ~2.4 kW/g for water colloids and ~1 kW/g for immobilized particles (AMF of ~63 kA/m and 309 kHz). The authors also showed that a reduction in the shell thickness or Co/Zn ratio favored the appearance of a collective magnetic behavior, arising from the competition between the dipolar and anisotropy energies of the CS_NPs. Such collective behavior led to the formation of chains in the colloid, which, according to the authors, was likely to be responsible for the large heating powers exhibited by their samples (see also Section 5).

Indeed, the magnetic heating mechanism has been studied in several types of CS_NPs, with different core size and/or shell thickness: Fe_3-x_O_4_ core (~4 nm) coated by a CoFe_2_O_4_ shell of variable thickness (1.0, 2.5, 3.5 nm) [109]; soft Fe_3_O_4_ core (varying between ~6 and 10 nm) and hard CoFe_2_O_4_ shell (varying between ~1 and 4 nm) [110]; Fe_3_O_4_ core (~6.3 nm) and CoFe_2_O_4_ shell (thickness 0.05, 1.0 and 2.5 nm) [111]; 14 nm sized NPs of Co ferrite core and a Mn-ferrite shell and inverted structure, with shells of varying thicknesses [112]; hard CoFe_2_O_4_ core and soft Ni_0.5_Zn_0.5_Fe_2_O_4_ shell (total size ~9 nm) [113]; hard CoFe_2_O_4_ core and soft AlFe_2_O_4_ shell (total size ~14 nm) [114]; Fe_3_O_4_ core and ZnCoFe_2_O_4_ shell and inverted structure (total size ~10 nm) [115].

CS_NPs with different CoFe_2_O_4_ core size (varying between ~4.4 and 8.4 nm), chemical nature of the shell (MnFe_2_O_4_ and iron oxide), and shell thickness (between ~1.9 and 3 nm) were prepared by Angotzi et al., using a seed-mediated growth strategy in an alcohol media and using an autoclave for heating [116]. In this case, initial cores were obtained by decomposition of metal oleates in a mixture of solvents (pentanol, octanol or toluene and water) in an autoclave at 180 or 220 °C/10 h and the shell was grown on the initial cores in a second reaction in the autoclave, adding the metal oleates in toluene, pentanol and water [117]. For all sets of samples, those with iron oxide shells featured higher heating efficiency and the thicker the soft shell, the better the performance.

In this framework, which highlights a general positive effect of hard/soft coupling in nanoheaters, perhaps the only exception is the article by Pilati et al. [118], dealing with Zn_x_Mn_y_Fe_z_O_4_/γ-Fe_2_O_3_ and Zn_x_Co_y_Fe_z_O_4_/γ-Fe_2_O_3_ CS_NPs. These CS_NPs were prepared by hydrothermal coprecipitation of metal salts in aqueous alkaline medium at 100 °C and the iron oxide shell was deposited onto them by precipitation of Fe(NO_3_)_3_. The authors elucidated how the chemical composition affected the saturation magnetization, the anisotropy and the heating properties of the CS_NPs. The two different sets of CS_NPs, having either hard or soft ferrite cores and soft maghemite shell, did not present evidence of any interfacial exchange coupling contribution to their power absorption efficiency.

**Figure 2:**
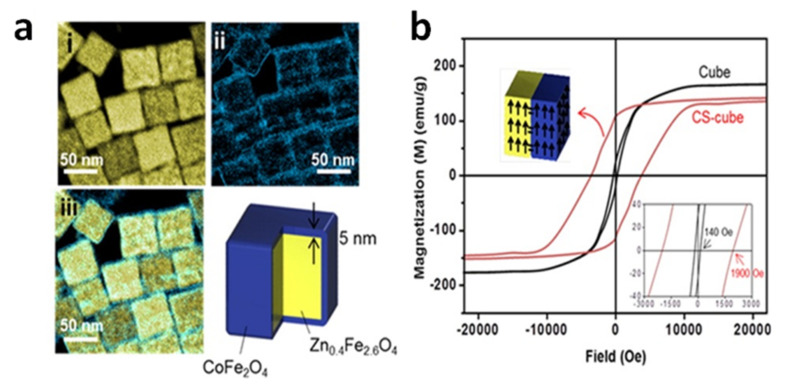
(**a**) Electron energy loss spectroscopy (EELS) mapped images of cubic CS_NPs with Zn_0.4_Fe_2.6_O_4_ core and CoFe_2_O_4_ shell. (i) Yellow and (ii) blue regions represent Fe and Co, respectively, and (iii) the merged image. (**b**) Magnetic hysteresis loops measured at T = 300 K on the CS_NPs and Zn_0.4_Fe_2.6_O_4_ cubic NPs with similar size (~60 nm). The coercivity of the CS_NPs was ~1900 Oe, 14 times larger than that of the single-phase cubes (~140 Oe). Adapted with permission from Ref. [119], Copyright 2012 American Chemical Society.

A heating efficiency as high as 10.6 kW/g (AMF of 37.3 kA/m and 500 kHz,) was measured by Noh et al. in cubic CS_NPs of Zn_0.4_Fe_2.6_O_4_ core (50 nm in edge) and CoFe_2_O_4_ shell (5 nm in thickness) [119]. The total size of the CS_NPs was ~60 nm and they did not exhibit superparamagnetic behavior (Figure 2). This high efficiency was attributed not only to the presence of the hard CoFe_2_O_4_ shell, but also to the cubic shape of the CS_NPs that, compared to the spherical shape, allowed to reduce magnetic disorder effects and hence to attain a saturation magnetization of the core closer to the value of the bulk phase.

As a matter of fact, the shape of the NPs has been proved to have a strong impact on the magnetic heating mechanism [120,121,122,123] and iron oxide nanocubes are among the best performing materials [52,124,125,126].

**Figure 3:**
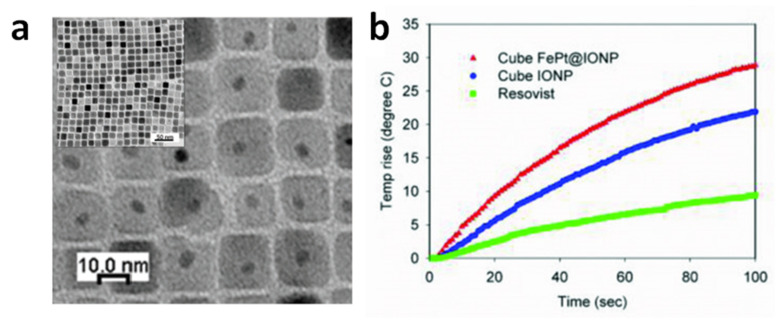
(**a**) Transmission electron microscopy (TEM) image of CS_NPs composed of a spherical FePt core embedded in a cube of Fe_3_O_4_. (**b**) Heating curves measured on the CS_NPs (sample FePt@IONP) and, for comparison, on single-phase magnetite cubic NPs (sample IONP) and on commercial iron oxide NPs (Resovist). Resovist and IONP showed SAR values of 0.39 and 0.92 kW/g, respectively, while the CS_NPs exhibited a value of 1.21 kW/g (AMF of 18.8 kA/m and 630 kHz). Adapted from Ref. [127] under CC BY_NC 4.0 International License.

The good synergy between cubic shape and exchange coupling was also demonstrated by Yang et al., who measured SAR = 1.21 kW/g in superparamagnetic CS_NPs composed of a spherical FePt core, of ~4.1 nm in size, embedded inside a cube of Fe_3_O_4_, so that the total size was ~14.7 nm (AMF of 18.8 kA/m and 630 kHz) (Figure 3) [127]. Again, we should emphasize that all these NPs showing high heating efficiency (SAR larger than 1 kW/g), were prepared by thermal decomposition in organic media, which is a method especially interesting for synthesizing CS_NPs with the low size dispersity, the good surface crystallinity and the homogeneous coating, required to maximize the coupling between the hard and soft phases. This method guarantees a strict control over the composition, structure, and morphology of the NPs. The main drawback of this technique is the difficulty of scaling up and the use of harmful and non-environmentally friendly reagents.

## 4. Multicore Nanoparticles (MC_NPs)

MC_NPs are sphere-like nanosized aggregates of magnetically interacting ‘primary’ NPs (i.e., the cores). Other terms can be found in literature to refer to this type of structures, such as clusters, nanoclusters, nanoassemblies and nanoflowers.

The magnetic interactions between the primary NPs, exchange or dipolar in type, improve their magnetothermal stability. Therefore, MC_NPs can exhibit either ferromagnetic behavior at room temperature or superparamagnetic relaxation, but at higher T_B_ compared to the constituent NPs (obviously, under the same measuring conditions). These two different possibilities depend on the sizes of the primary NPs and of the final aggregated structure and also on the type and strength of magnetic interactions between the primary NPs, which in turn are determined by their spatial arrangement. All these factors ultimately depend on the chemical synthetic method, which determines the reaction rate and the degree of fusion between the cores [128]. Thus, depending on the viscosity of the media, the temperature and the heating time, multicore structures made of individual random cores or well-oriented cores can be obtained.

In a very large number of articles on magnetic NPs, the absence of magnetic hysteresis, usually ascertained by DC magnetometers (typically SQUID or vibrating sample magnetometer, VSM), is considered as an evidence that the NPs are in the superparamagnetic regime. In our opinion, although the measurement of null values of H_C_ and M_r_ strongly supports this interpretation, it does not constitute definitive proof. Using DC magnetometry, a complete study of the magnetic relaxing behavior of the NPs should include the measurement of hysteresis loops at different temperatures and the evaluation of the anisotropy energy barrier distribution, and hence of the T_B_ distribution, through the analysis of the thermal dependence of the thermoremanent magnetization or of the zero-field-cooled (ZFC) field-cooled (FC) magnetization [39,71]. Particularly in the case of magnetic NPs that tend to form spherical aggregates, it is not uncommon to measure null values of H_C_ and M_r_ at a temperature significantly lower than the higher T_B_ of the assembly assessed by ZFC-FC magnetization measurements [129]. The possible explanation is that the moments of the NPs arrange in low-remanence flux-closure configurations [55,130,131,132]. This occurs in order to minimize the energy of the aggregate as a whole, reaching a balance between the magnetostatic energy, the anisotropy energy and the contribution of the magnetic interactions between the NPs, all of which vary with temperature due to the thermal dependence of M_S_ and K. Therefore, one can observe that both M_r_ and H_C_ decrease strongly on increasing temperature and possibly become smaller than the measuring experimental error.

As for MC_NPs, the onset of low-remanence magnetic configurations is certainly not favorable as regard the heating efficiency and therefore it would deserve attention. On the other hand, regardless of the real reason behind the absence of magnetic remanence at room temperature, this feature has been considered a point of strength of this type of nanostructures, just as it is for single-core superparamagnetic NPs [133]. In fact, a null or small remanence implies that dipolar interactions between the MC_NPs are suppressed or strongly decreased. This reduces the risk of formation of large agglomerates, in the micrometer range, that may occlude blood vessels of a patient and cause dangerous side effects. Regarding biomedical applications, the relatively large size of MC_NPs favors high cellular uptake and prolonged circulation in the blood stream [134,135,136].

In general, ascertaining whether the magnetic behavior of MC_NPs is dominated by exchange or dipolar interactions is not simple and in some articles the item is not explored in detail, actually. However, exchange and dipolar interactions may influence the overall magnetic properties of spherical aggregates differently depending on their nature, which is magnetizing and demagnetizing respectively. In Section 4.1 and Section 4.2, we will try to better highlight the role of exchange and dipolar interactions and we will present some selected examples taken from literature, also on the basis of the scientific novelty they represented.

Different synthesis methods have been described for the preparation of iron oxide MC_NPs with sizes between 20–250 nm, starting from Fe(II), Fe(III) or a mixture of both, in organic, aqueous and polyol media, using different surfactant to control the reaction rate and finally, using different heating sources, microwave, autoclaves or a heating mantle [128]. It is worth mentioning that, when the synthesis is performed in aqueous media, large aggregates with poor internal order are generally obtained. When the synthesis is in organic media, multicore structures have been reported as intermediate steps, with a certain degree of internal order [137]. However, when the synthesis takes place in polyol media, the adsorption of solvent molecules on the primary cores causes in situ-oriented aggregation [138]. It has been shown that polyol molecules are mainly adsorbed on some specific faces, favoring intermolecular hydrogen bonding from one covered core to another. Interestingly, at high surface coverage, polyol molecules are detached leading to a highly ordered multicore nanostructure.

In fact, one of the most used methods for preparing MC_NPs with internal exchange interactions (Section 4.1) is the synthesis in polyol media because of its versatility and reproducibility [139]. The polyol is a polar media to dissolve the precursors and determine the maximum temperature of heating. Then, there is a biocompatible complexing agent, that can be polyacrylic acid (PAA) or polyethylene glycol (PEG), and a base, sodium hydroxide, sodium acetate or an amine, that initiates the nucleation of the precursors and form the initial cores. Then, these cores are immediately aggregated into three dimensional nanostructures, stabilized with the excess of the complexing molecules.

MC_NPs around 40–50 nm were obtained by the polyol method using iron(III) acetylacetonate as precursor, tri(ethylene glycol) (TREG) and triethanolamine (TREA) heated to reflux and maintained at the refluxing temperature (245–280 °C) under the argon flow for 1 h [140]. Increasing the amount of base concentration, the nucleation of the iron oxide NPs became faster, so that the nucleated particles tried to aggregate to reduce their surface energy. By a similar method, but using diethylene glycol (DEG), Mn-doped iron oxide MC_NPs of 50 nm were prepared [141]. This method allowed the successful incorporation and homogeneous distribution of Mn within the MC_NPs. Slightly smaller sizes, 24–29 nm, were obtained by using a mixture of glycols, diethylene glycol (DEG)/tetraethyleneglycol (TEG) in a heating mantle up to 250 °C [142]. By using a microwave-assisted polyol approach Fe_2_(SO_4_)_3_, sodium acetate and PEG in EG, MC_NPs of 27 to 52 nm could be obtained at 200 °C, increasing the size with the increase in reaction time from 10 s to 600 s [143]. Much larger MC_NPs (<250 nm) were be obtained by using an autoclave assisted polyol method, starting from Fe(III) in EG, urea and PAA, composed of many Fe_3_O_4_ nanocrystals with size < 10 nm [144].

Clustering previously synthesized primary NPs is the preferred route for obtaining MC_NPs with internal dipolar interactions (Section 4.2). First, individual NPs are obtained, either in water or in organic media, coated with oleic acid and then they are aggregated in a second step, using either citric acid, for example for the hydrophilic ones, or an oil in water emulsion, using a surfactant such as CTAB [145]. In general, these methods involving synthesis and aggregation of NPs yield non-uniform and large size distributions, having mainly dipolar magnetic interactions between cores.

### 4.1. MC_NPs with Internal Exchange Interactions

Exchange interactions are possible in multicore structures when the primary NPs are in a very close contact, substantially fused together. The terms ‘grains’ or ‘nanocrystals’ are also used instead of primary NPs. This condition of intimate and extensive structural connection is usually accompanied by a good degree of crystallinity of the grains and hence a high saturation magnetization, comparable to that of bulk materials. In fact, surface and structural/magnetic disorder effects that mostly affect single-core ferrite NPs, such as an alteration of the spinel structure and spin-canting [146,147,148,149,150,151], are strongly reduced.

In bulk nanocrystalline magnetic materials, the exchange interaction tends to couple ferromagnetically the atomic spins of neighboring nanocrystals, in competition with their local magnetic anisotropy. This can cause a reduction of the effective magnetic anisotropy of the system, compared to that of the individual nanocrystals, and hence of the coercivity. If the size of the nanocrystals is comparable to their ferromagnetic exchange correlation length, the anisotropy decrease is restrained [152]. On the other hand, if the nanocrystal size is lower than the ferromagnetic exchange correlation length, the local magnetic anisotropy is substantially averaged out to zero, which leads to superior soft magnetic properties [153]. Being magnetizing in nature, exchange interactions result in high susceptibility and favor the remanence of the magnetization.

Passing to MC_NPs, it must be considered that the magnetic properties and their thermal evolution, including the magnetization configuration, are determined by the interplay between this exchange-coupling phenomenology and small-size effects, i.e., magnetic relaxation and magnetostatic effects. The examples here below demonstrate that this interplay can actually lead to excellent heating capacity.

**Figure 4:**
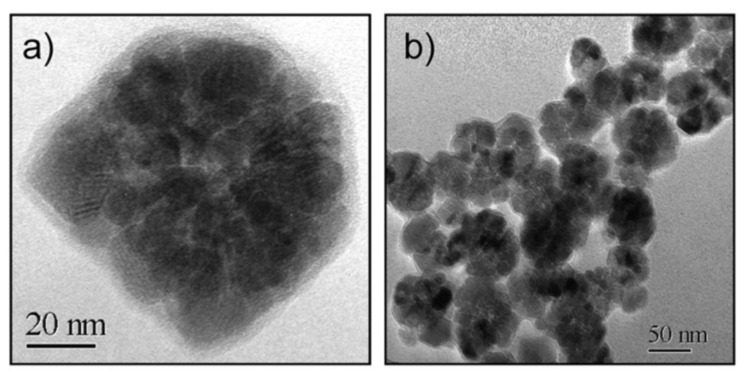
Typical TEM images of (**a**) a MC_NP consisting of exchange-coupled iron oxide cores and (**b**) an ensemble of MC_NPs. Reused with permission from Ref. [154], Copyright 2009 Elsevier B.V. All rights reserved.

In a 2009 article, Dutz and coworkers stressed the suitability of MC_NPs for biomedical applications, particularly magnetic hyperthermia and cell separation [154]. The authors reported about water-based suspensions of aggregates of ~40–80 nm (coated by a carboxymethyldextran shell) consisting of primary iron oxide NPs with mean size of 14 nm (Figure 4). The synthesis was carried out by coprecipitation in aqueous media (100 °C). The material showed ferrimagnetic behavior owing to the exchange interaction between the cores [133]. The highest SAR ~ 330 W/g was measured in MC_NPs with hydrodynamic size of ~82 nm (AMF of 11 kA/m and 400 kHz).

In the same year, Barick et al. prepared Fe_3_O_4_ spherical MC_NPs of ~40 nm in size by the polyol method (EG, 200 °C) [155]. The MC_NPs were porous and composed of highly crystalline primary NPs of ~6 nm, which, as observed by TEM, were pseudoepitaxially fused together. At T = 300 K, the MC_NPs showed a higher magnetic susceptibility, compared to that of 6 nm Fe_3_O_4_ NPs taken as reference, and a much higher magnetization (~64.3 emu/g under a static field of 20 kOe). This behavior was explained in terms of a collective magnetic behavior of the primary NPs, induced by exchange coupling and dipolar interactions. Although we agree that, due to the porous nature of the MC_NPs, both types of magnetic interactions could be active, the increased susceptibility and magnetization seem more consistent with predominant exchange interactions, in our opinion. No magnetic hysteresis was observed at T = 300 K and the authors concluded that the MC_NPs were superparamagnetic. Although the article was mainly focused on the excellent properties shown by these nanostructures as contrast agents in Magnetic Resonance Imaging (MRI), the ability to generate heat under an AMF was also demonstrated (SAR = 92.62 W/g_Fe_ in AMF of 10 kA/m and 425 kHz).

**Figure 5:**
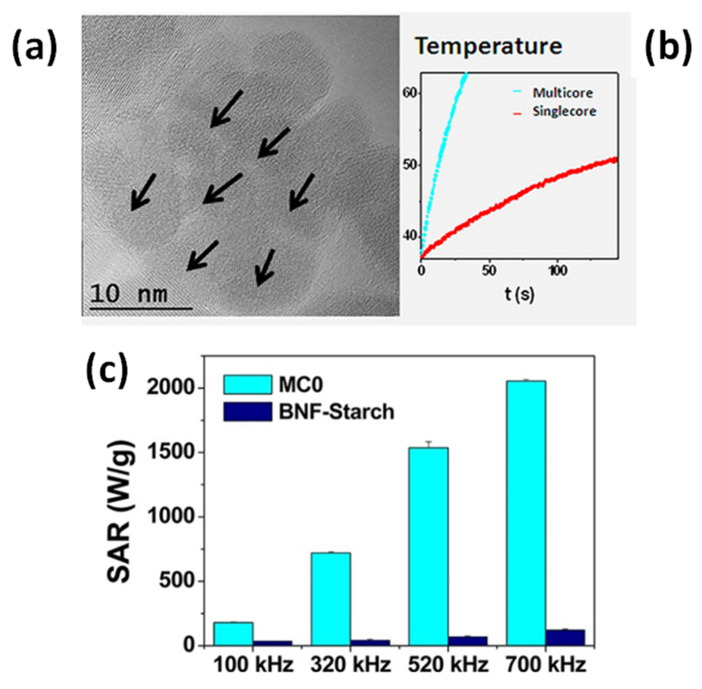
(**a**) High resolution TEM image of a maghemite MC_NP consisting of merged grains with the same crystalline orientation. (**b**) Heating curves measured on the MC_NPs and on single-core NPs (AMF of 29 kA/m and 520 kHz). (**c**) SAR value comparison between the MC_NPs (sample called MC0) and commercial magnetite NPs (BNF_starch) at different frequencies for a magnetic field amplitude of 25 kA/m. Adapted with permission from Ref. [156], Copyright 2012 American Chemical Society.

Lartigue et al. prepared maghemite MC_NPs through a polyol process similar to the previous one (TEG, 220 °C) [156]. In particular, they obtained citrate-stabilized nanostructures ranging from single-core NPs of 10 nm to MC_NPs, with different mean size (19.6, 22.2, 24 and 28.8 nm). Transmission electron microscopy (TEM) techniques revealed that the MC_NPs consisted of merged grains sharing a same facet, namely the grains had the same crystalline orientation and the continuity of the crystal lattice at the grain interfaces could be clearly observed (Figure 5a). The saturation magnetization of the MC_NPs was close to that of bulk maghemite, unlike that of single-core NPs which was 30% lower, while the magnetic anisotropy was reduced (1.75 ÷ 2.5 × 10^4^ J/m^3^ for MC_NPs and 2.6 × 10^4^ J/m^3^ for single-cores). The MC_NPs were superparamagnetic at room temperature, as observed by SQUID, but the blocking temperature T_B_ was considerably higher than that of the single-cores. Based on the whole of experimental results, the authors hypothesized that the cores were exchange interacting. Heating tests were carried out for different AMF amplitudes (9 ÷ 29 kA/m) and frequencies (100 ÷ 700 kHz). Under all conditions, a 2 ÷ 10-fold SAR increase was observed for MC_NPs with respect to single-cores. In AMF of 29 kA/m and 520 kHz, the largest sized MC_NPs produced the highest SAR (above 1.5 kW/g) (Figure 5b,c). The authors concluded that the combination of reduced anisotropy and enhanced magnetic moment, made possible by magnetic ordering and exchange interactions at the grain interfaces, preserved the superparamagnetic-like behavior of the MC_NPs and simultaneously increased the thermal losses.

This work by Lartigue at al. was published shortly after another article by the same group, in which the authors described in detail the polyol synthetic protocol and discussed the formation mechanism of maghemite MC_NPs, with variable size, made up of grains of approximately 11 nm [157]. The article highlighted the great heating capacity of this type of structures and for MC_NPs of 24 nm a SLP value as high as ~2 kW/g was reported (AMF of 21.5 kA/m and 700 kHz). In this article, the authors did not mention the possible role of exchange interactions and the exact heating mechanisms was not delineated, actually. However, they already observed that the MC_NPs were single crystalline and that, probably thanks to the high crystallinity degree, had a high saturation magnetization, close to that of bulk maghemite or, in some samples, even the same.

**Figure 6:**
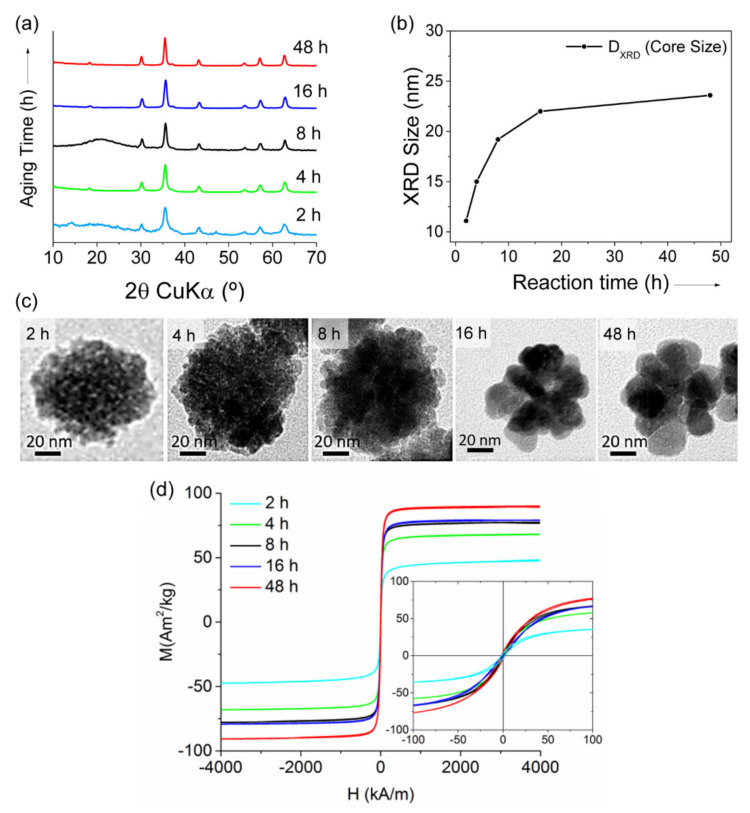
Structure of maghemite MC_NPs at different reaction time (2–48 h) during the synthesis with polyol method: (**a**) X-ray diffraction patterns; (**b**) core size calculated by Scherrer’s equation. (**c**) Representative TEM images. (**d**) Magnetization curves measured on the samples at T = 290 K: M_S_ increased from 48 to 90 Am^2^/kg with prolonging the reaction time, as well as H_C_ (0.5−2 kA/m). Adapted with permission from Ref. [158], Copyright 2017 American Chemical Society (further permissions related to the material excerpted should be directed to the ACS).

The polyol method was also used by Gavilán et al. starting from an Fe(III) salt to prepare maghemite MC_NPs of ~60 nm, but with different size of the cores [158]. In fact, on increasing the reaction time, the MC_NPs undertook a crystallization process that increased the core size from ~11 nm to ~23 nm, as well as the saturation magnetization (Figure 6).

An enhanced magnetic susceptibility and a smaller coercivity, compared to that of 36 nm single-core NPs, indicated a collective magnetic behavior of the constituent primary NPs. The highest SLP (~1.13 kW/g_Fe_ in AMF of 23.8 kA/m and 710 kHz) was measured in water suspension of MC_NPs with the largest core size (i.e., 23 nm). This value was 5 times larger than that of MC_NPs with cores of 15 nm and 1.5 times larger than the 36 nm single-core NPs. However, a drastic decrease (~37%) of the heating performance of the MC_NPs tested in a viscous medium (agar 2%) was reported.

Indeed, a similar effect was also observed for 25 nm MC_NPs, similar to those of Refs. [156,157], by comparing heating tests in water and in high viscosity glycerol [159]. The authors reported an even stronger decrease of the heating efficiency (up to 90%) for MC_NPs in cellular environment (i.e., attached to the cell membrane or internalized within intracellular vesicles), though this result seems somewhat contradictory to the previously reported good ability of these nanostructures to kill breast cancer cells under AMF exposition [156]. It was argued that the heating reduction was due to the inhibition of the Brownian mobility in glycerol and in the cells, caused by the high viscosity.

In fact, in MC_NPs, the magnetization by Brownian motion may be relevant due to the large magnetic moment they can acquire in an applied field. However, particularly in the case of dispersion in biological media or confinement in cells, aggregation states can be favored and hence dipolar interactions can come into play, which affect the heating mechanism [160,161,162]. This point is further addressed in Section 4.2 and Section 6.

Bender et al. studied the hyperthermia performance of dextran coated maghemite MC_NPs, of about 39 nm in size, constituted by exchange-coupled 5−15 nm cores and hydrodynamic sizes (z-average) of 56 nm with a polydispersity index of 0.099 [163]. The heating tests were carried out on the colloidal dispersion whose viscosity was changed by adding glycerol (AMF of 8.8 mT and rotational frequency ϖ = 5.9 × 10^6^ Hz). Considerably high ILP values were measured (~7 nHm^2^/kg_Fe_) and nearly independent of the viscosity, indicating that, at this high AMF frequency and under the adopted experimental conditions, the heat was generated by internal magnetization processes and not by Brownian relaxation.

Hence, the problem of the efficiency of MC_NPs in highly viscous media, including cells, is complex, somewhat controversial, but certainly can be a concern. It depends in part on the existence of remanence in these multicore structures or on the poor colloidal stability in different media that may lead to agglomeration of the material. On the other hand, several articles have confirmed the excellent suitability of these nanostructures as hyperthermia agents in cancer treatments.

Dutz et al. performed heating tests (AMF of 25 kA/m and 400 kHz) on maghemite MC_NPs of 40–60 nm in size dispersed in fluid (SAR = 400 W/g) and embedded in gelatin, i.e., immobilized as in a tumor tissue (SAR = 262 W/g) [164]. In spite of the SAR reduction, in vivo experiments in mice demonstrated that these MC_NPs heated a tumor of about 100 mg by about 22 °C within the first 60 s of treatment.

Hemery et al. compared the efficiency of iron oxide MC_NPs (29.1 nm) and single-core NPs (14.5 nm) for magnetic hyperthermia treatments on glioblastoma cells [134]. The samples were produced by a polyol method [165] and for this study the authors selected samples with SAR of 265 W/g and 134 W/g for the MC_NPs and for the single-core NPs, respectively (AMF of 10 kA/m and 755 kHz). The study highlighted the superior efficiency of MC_NPs for magnetic hyperthermia, leading to 80% cancer cell death, which was ascribed to the higher SAR and better cellular uptake.

Shaw et al. synthesized MC_NPs with size around 40–60 nm consisting of γ-Fe_2_O_3_ grains grown over the surface of a MnFe_2_O_4_ core [166]. Microwave-assisted polyol method in two steps was used to obtain first the MnFe_2_O_4_ seed and then the MnFe_2_O_4_/γ-Fe_2_O_3_ structure. The exchange interaction within the MC_NPs led to enhanced M_S_ and magnetic susceptibility, compared to MnFe_2_O_4_ cores alone. A magnetic hyperthermia treatment carried out for 30 min on HeLa cells, with 0.75 mg/mL ferrofluid of the MC_NPs, induced a temperature rise to 46 °C and decreased the cell viability to 17% (AMF of 250 Oe and 113 kHz).

An original type of 100 nm MC_NPs, made of Fe_0.6_Mn_0.4_O (wüstite), were synthesized by Liu et al. by thermal decomposition of acetate precursors in trioctylamine and PEG (Mw = 10,000) to make them hydrophilic by ligand exchange reaction [167]. The MC_NPs exhibited unique room-temperature ferromagnetic behavior, unlike their antiferromagnetic bulk counterpart, thanks to the high iron content and to the exchange coupling between the cores that enhanced the ferromagnetic ordering. Heating tests (AMF of 40 mT and 366 kHz) gave SAR = 535 W/g_Fe_ in aqueous solution and 490 W/g_Fe_ in agarose gel. In vitro and in vivo magnetic hyperthermia experiments demonstrated that these wüstite MC_NPs could induce breast cancer cell apoptosis and a complete tumor regression in tumor-bearing mice without appreciable side effects.

Similar results were obtained for cRGD coated 20 nm manganese iron oxide MC_NPs obtained by similar method, with maximized SAR (680 W/g at moderated AMF of 47 kA/m and 96 kHz,) and very efficient results in a human tumor-derived glioblastoma cell line U87MG (62% cell death) [168].

### 4.2. MC_NPs with Internal Dipolar Interactions

If the cores forming the MC_NPs are not enough closely packed or if the intimate contact between them is not extended or is prevented by a non-magnetic coating, dipolar interactions predominate.

The influence of dipolar magnetic interactions on the magnetothermal behavior of a NP assembly has been extensively studied in the last decades and different, sometimes conflicting, models have been applied to explain the experimental data [39,169,170]. In general agreement with numerical calculations and theoretical predictions [171,172], we could summarize the role of dipolar magnetic interactions acting on a NP assembly by saying that they produce two main competing magnetic effects, relevant for the magnetic heating mechanism. As formerly indicated by Dormann et al. [39], dipolar magnetic interactions lead to an increase of the anisotropy energy barriers of the NPs. In the case of small and soft NPs, this effect improves the thermal stability of their magnetic moments, shifting to higher temperature or preventing the entrance in the superparamagnetic regime [39,129,149,173,174,175,176]. Under the conditions of validity of the LRT (i.e., in the linear regime), dipolar interactions can vary the Néel relaxation time τ_N_ so as to approach the resonant condition f_m_τ_N_ = 1 or move away from it, which leads to an increase or decrease of the hysteresis loop area, respectively; in the nonlinear regime, increasing τ_N_ so as to pass from the superparamagnetic state (f_m_τ_N_ < 1) to the blocked one (f_m_τ_N_ > 1) increases more and more the hysteresis loop area, at least until demagnetizing effects become prevalent [172]. In fact, in blocked NPs, magnetic dipolar interactions exert a demagnetizing action and bring about a decrease of remanent magnetization, magnetic susceptibility and possibly of coercivity. Both experimental and modeling results have confirmed this second effect of dipolar interactions, which is clearly detrimental to the heating efficiency [171,177,178,179,180,181,182].

Hence, the role played by dipolar interactions in the heat generation mechanism is complex: while increasing the effective anisotropy of the NPs can enhance the heating efficiency [175,183,184,185], their demagnetizing nature is disadvantageous. Therefore, the final heating performance comes from the competition between these different effects, which ultimately depends on the specificities of the system, i.e., size and anisotropy of the individual NPs and aggregation state, and on the measurement conditions (AMF amplitude and frequency) [171]. Some experiments and numerical simulations revealed a non-monotonic evolution of SAR on increasing the concentration of ferrofluids, hence the strength of dipolar interactions, on a wide range of values [176,186,187,188,189]. It was highlighted the existence of a SAR peak at an optimal concentration at which dipolar interactions are comparable to the anisotropy field [186,188].

When dipolar interactions give rise to stable aggregates of NPs, configurational effects of magnetostatic nature must be also included in this description. In isometric aggregates of soft NPs (i.e., spherical aggregates, precisely what we call MC_NPs) a low-remanence magnetic state is favored, which implies a decreased heating efficiency (in the case of hard NPs, their individual anisotropy dominates on dipolar interactions); on the opposite, anisometric aggregates (i.e., elongated formations such as chains and columns) exhibit high-remanence magnetic configurations, which can enhance the heating efficiency (see Section 5) [55]. Moreover, a reduced Brownian mobility connected to an increased hydrodynamic size may also enter into this already intricate picture [190].

Therefore, MC_NPs with internal dipolar interactions do not necessarily guarantee better heating efficiency than individual NPs. The main advantage of this type of nanostructures is represented by the possibility of controlling their structural characteristics (such as size of the cores, distance between the cores, size of the aggregates), hence the state of magnetic interaction and the heating capacity. In this regard, some examples are now shown.

Blanco-Andujar et al. reported on the heating properties of citric acid coated iron oxide MC_NPs, obtained by coprecipitation in a microwave reactor and coated in a second step [191]. This particular synthesis method allowed to control the size (varying between 13 and 17 nm) and number of the individual magnetic cores and hence the hydrodynamic size D_H_ of the aggregated structure (50–125 nm), by changing the time of heating in the second step. The samples did not show magnetic hysteresis at room temperature in SQUID measurement conditions. The existence of inter-cores demagnetizing interactions of dipolar type was verified through the analysis of the field dependence of the remanence (isothermal remanent magnetization, IRM, and direct current demagnetization, DCD) and the construction of the Henkel plots. A better heating efficiency was associated with a lower core-to-core magnetic interaction. The best response (ILP of 4.1 nHm^2^/kg) was measured for small MC_NPs (D_H_ ~ 65 nm) consisting of large cores (~17 nm).

Sakellari et al. studied the heating properties of colloidal MC_NPs of various size (45–98 nm), consisting of 13 nm iron oxide NPs prepared by the polyol process in the presence of PAA. Water content seems to be the parameter to control the multicore size [192]. The packing density of the MC_NPs increased with the size and therefore the saturation magnetization increased too. In spite of the small size of the primary NPs, the samples showed ferrimagnetic behavior at room temperature, which was ascribed to an enhanced blocking temperature T_B_ due to dipolar interactions [193]. The thermal response of the MC_NPs was higher than that of the individual NPs. The 50 nm MC_NPs showed the best heating capacity (maximum SAR ~ 400 W/g in AMF of 25 kA/m and 765 kHz) as a result of the optimized interplay between structural features, packing density and strength of dipolar interactions.

Ovejero et al. studied the effects of dipolar interactions in iron oxide MC_NPs prepared by thermal decomposition in organic media, transferred to water by ligand exchange with DMSA and controlling aggregation by changing the pH of the dispersion [189]. The primary NPs had a hydrodynamic size D_H_ = 20 nm. The D_H_ of the MC_NPs varied between 56 nm and 356 nm. The authors stressed the influence of the polydispersity index (PDI) in the heating mechanism. In fact, the heating capacity was strongly influenced by the dipolar interactions resulting from the aggregation of the NPs, but in a different manner depending on PDI. For low PDI (<0.2), the SAR value slightly increased at small values of D_H_ (<100 nm) and then showed a 25% drop starting from D_H_ =139 nm. For high PDI, a progressive but smooth reduction of SAR (~10%) was observed on increasing D_H_. Thanks to the analysis of high frequency hysteresis loops, the authors also provided some hints on the causes of such SAR dependence. The increase of dipolar interactions due to the increase of aggregation state resulted in a reduction of the remanent magnetization, though accompanied by an enhancement of coercivity.

**Figure 7:**
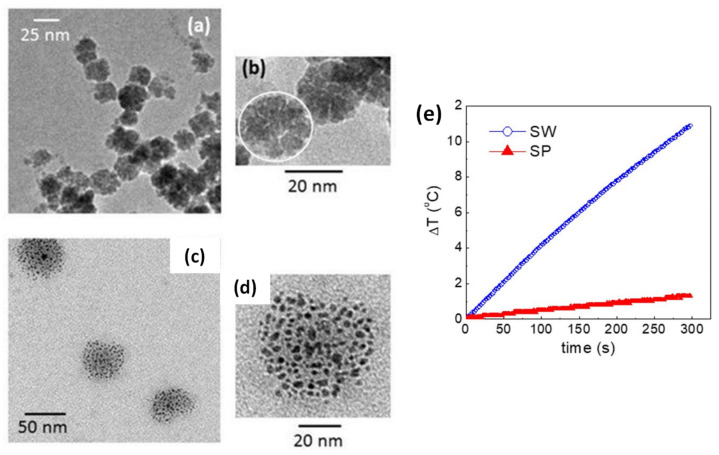
(**a**,**b**) TEM images of iron oxide MC_NPs dispersed in water (sample SW); in (**b**) the white circle highlights one MC_NP consisting of primary NPs capped with 2-pyrrolidone. (**c**,**d**) TEM images of the MC_NPs dispersed in polyethylene glycol 400 (PEG) (sample SP), showing how the primary NPs tended to separate from each other. (**e**) Heating curves for samples SW and SP in an AMF of 13.5 kA7m and 177 kHz. Adapted from Ref. [151] under CC BY 4.0 License.

Another strategy for controlling the dipolar interactions of MC_NPs was presented by Spizzo et al., who prepared aggregates, of ~25 nm in size, consisting of small iron oxide NPs (5–10 nm) with 2-pyrrolidone as capping agent [151]. Thanks to the presence of 2-pyrrolidone, the MC_NPs were stable in water. Conversely, when dispersed in PEG, the primary NPs tended to separate from each other, although they still formed spherical aggregates (Figure 7a–d). Thus, the strength of the dipolar interactions between the primary NPs varied upon changing the fluid in which they were dispersed. Accordingly, the heating response varied too and, in AMF of 13.5 kA/m and 177 kHz, the best response was measured in the aqueous dispersion of MC_NPs, namely in the more compact structures dominated by stronger dipolar interactions (Figure 7e).

**Figure 8:**
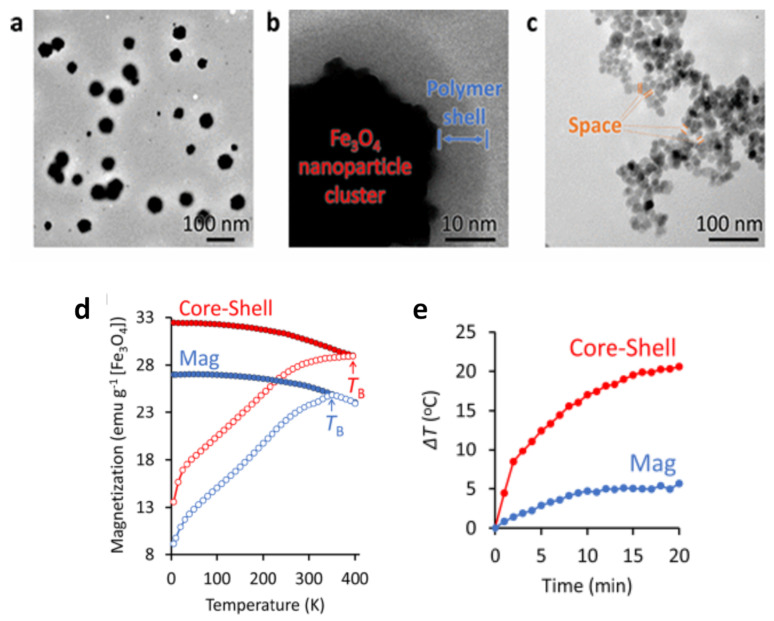
(**a**) TEM image of magnetite MC_NPs (**b**) Magnified view of a MC_NP allowing to distinguish the existence of a 6 nm-thick polymer shell. (**c**) TEM image of unclustered Fe_3_O_4_ NPs, prepared as control material. (**d**) ZFC-FC magnetization measurements vs. T measured on the MC_NPs (indicated as core-shell sample due to the presence of the polymer coating) and on the control NPs (sample called Mag). (**e**) Changes in the temperature of an aqueous solution containing MC_NPs and the control NPs with AMF application time. Adapted with permission from Ref. [194], Copyright 2016 American Chemical Society (further permissions related to the material excerpted should be directed to the ACS).

To produce tight clustering of Fe_3_O_4_ NPs and highlight the importance of the primary NPs in cluster formation for enhanced heat-generation power, Hayashi et al. prepared magnetite NPs (~17 nm in diameter) by precipitation with hydrazine in the presence of a pyrrole polymer that formed multicore structures of ~55 nm, also containing anticancer drug (i.e., doxorubicin, DOX) [194]. NP size was controlled by adjusting the amount of hydrazine and the reaction time. Folic acid and PEG were used to stabilize the multicore structures in suspension, leading to final MC_NPs of (64 ± 6) nm with ferrimagnetic behavior at room temperature (Figure 8a–c). In fact, strong dipolar interaction between the primary NPs influenced the passage to the superparamagnetic regime, which, using SQUID magnetometry, was seen to occur at T_B_ ~ 400 K. For comparison, in a sample of unclustered Fe_3_O_4_ NPs, prepared as control material, T_B_ ~ 350 K (Figure 8d). A maximum SAR value of 353 W/g was measured in the MC_NPs, more than double that of the control NPs (AMF of 8 kA/m and 217 kHz) (Figure 8e). The capacity of the MC_NPs to hold the DOX and the possibility to control its release using the AMF as a trigger was also investigated. This work followed previous articles by Hayashi and co-workers, which were also aimed at demonstrating the suitability of Fe_3_O_4_ MC_NPs as theranostic agents [23,195].

Regarding the potential of MC_NPs as multifunctional agents in nanomedicine applications, it is worth highlighting the high efficiency of these nanostructures as MRI contrast agents. The topic has been dealt with in a number of articles and the *T*_1_ and *T*_2_ contrast enhancement by aggregation of iron oxide NPs is a well-established phenomenon [140,196,197,198,199], common to MC_NPs dominated both by exchange interactions [155,156,165,167] and dipolar interactions [141,193,194,195].

## 5. Linear Aggregates

With the term ‘linear aggregates’, we refer to anisometric assemblies of NPs, both 1-dimensional (i.e., chains) and 3-dimensional (columnar aggregates), coupled by dipolar interactions. The research interest in the magneto-heating properties of this kind of magnetic structures has been prompted by two main factors: the discovery of the excellent heating performance exhibited by chains of magnetosomes synthesized by magnetotactic bacteria [200,201,202,203] and the observation that magnetic NPs in a fluid tend to arrange in linear aggregates under a uniform magnetic field (static or alternating).

Magnetosomes consist of a highly crystalline cubic-shaped core of magnetite/maghemite surrounded by biological materials, in particular lipids and proteins. Typically, the cores have a mean size of ~30 nm, are single magnetic domains and show ferrimagnetic properties at room temperature. The magnetosomes are naturally arranged in chains inside the bacteria thanks to protein filaments that favor their alignment [204]. It must be noted that, in the chain configuration, the dipolar interactions favor the ferromagnetic alignment of the moments of the NPs, i.e., they exert a magnetizing action. This marks a fundamental difference with respect to isometric aggregates of randomly oriented NPs, where dipolar interactions are demagnetizing, as discussed above (Section 4.2).

It was argued that the superior magnetic efficiency of magnetosomes was only in part due to the cubic shape of the individual cores—which implied a higher surface magnetic anisotropy, compared to spherical iron oxide of similar size—and that the chain arrangement was a crucial element [126]. In fact, dipolar interactions between the cores was found to result in an effective magnetic anisotropy of configurational type [204].

The onset of an effective anisotropy, induced by the formation of linear aggregates during heating tests and oriented parallel to the applied field, was also invoked to correctly interpret magnetic heating performance of NPs in fluids [55,58]. This effective uniaxial anisotropy is substantially the shape anisotropy of the whole aggregate and competes with the magnetocrystalline anisotropy of the individual NPs. Therefore, it is more effective in the case of low anisotropy NPs, where it leads to an increase of the hysteresis loop area and hence of the SAR [55].

Excluding a few theoretical studies that reported a decrease in heating efficiency related to NP chain formation [205,206], in general both theoretical analyses and experimental results confirmed that chain-like arrangements of NPs had enhanced heating performance compared to systems of randomly distributed NPs and pointed out a dependence on the features of the linear aggregates (width, length and density as well as the size and shape of the constituent NPs) [57,207,208,209]. In the latter cited articles, samples of linear aggregates suitable for heating tests were prepared by quenching magnetic NPs in an agarose gel matrix in the presence of a static uniform magnetic field, so as to develop anisotropic dipolar interactions between them. Hence, it was also shown that the heating efficiency of these systems could be modified by changing the viscosity of the agarose matrix and the relative orientation between the aggregate long axis and the direction of the AMF [207,208,209]. This last item was also theoretically addressed by Valdes et al., who also analyzed the case of randomly oriented NP chains and predicted significantly better heating performance with respect to a system of dispersed non-interacting NPs [210,211].

The better heating capacity of anisometric groups of NPs was also demonstrated by Niculaes et al. who compared the SAR values of individual iron oxide nanocubes (edge length ~20 nm) of dimers and trimers (composed of two and three nanocubes, respectively) and of larger aggregates of more than four nanocubes [212]. The highest SAR was measured in the anisometric dimers and trimers whereas the larger and more isometric structures exhibited the lower thermal response.

Avugadda et al. fabricated aggregates of iron oxide nanocubes, coated with a bioresorbable polymer, that could be disassembled upon exposure to lytic enzymes, thus obtaining 2D assemblies and finally small chain-like clusters, containing just few nanocubes, with improved heating performance [213].

Balakrishnan et al. showed that cubic-shaped cobalt ferrite NPs (mean edge size ~17 nm), injected in tumors developed in mice, spontaneously formed randomly oriented chain-like structures (median of 4 nanocubes/chain), whose length increased (median of 7 nanocubes/chain) after exposure to an AMF during an heating treatment [214].

Fu et al. prepared compact aggregates of magnetite NPs of different sizes, using an emulsion droplet solvent evaporation method [215]. They showed that dipolar interactions between the NPs in the aggregates improved the heating efficiency as long as the latter were small and anisometric, so as to favor the appearance of shape magnetic anisotropy. As the size of the aggregates increases, they became more and more spherical. Thus, the shape anisotropy decreased and this impaired the heating efficiency.

As for the production of long linear aggregates of NPs, excluding the possibility of extracting magnetosome chains from cultured magnetotactic bacteria [201], the adopted methods involve the use of an externally applied magnetic field, as already seen above for immobilization into an agar matrix. In this respect, another example is the work of Hu et al., who inserted linear aggregates of NPs, of 15 nm and 200 nm in size, in a hydrogel matrix [216]. This was obtained by assembling the magnetic NPs in monomers solution and then activating the gelation, in presence of a magnetic field during both processes.

The use of an inorganic shell-like silica to encapsulate linear arrangements was also explored by Andreu et al. [217] to produce small chains of cubic iron oxide NPs mimicking naturally produced magnetosomes and enhance the SAR respect to individual nanocubes. Comparing these chains with individual nanocubes for a fixed AMF of 3 kA/m and 111 kHz, they observed that the heating performance of chains resulted higher at room temperature, but lower at low temperatures (<250 K). These results stress the importance of considering the temperature and AMF conditions for comparing the heating efficiency.

**Figure 9:**
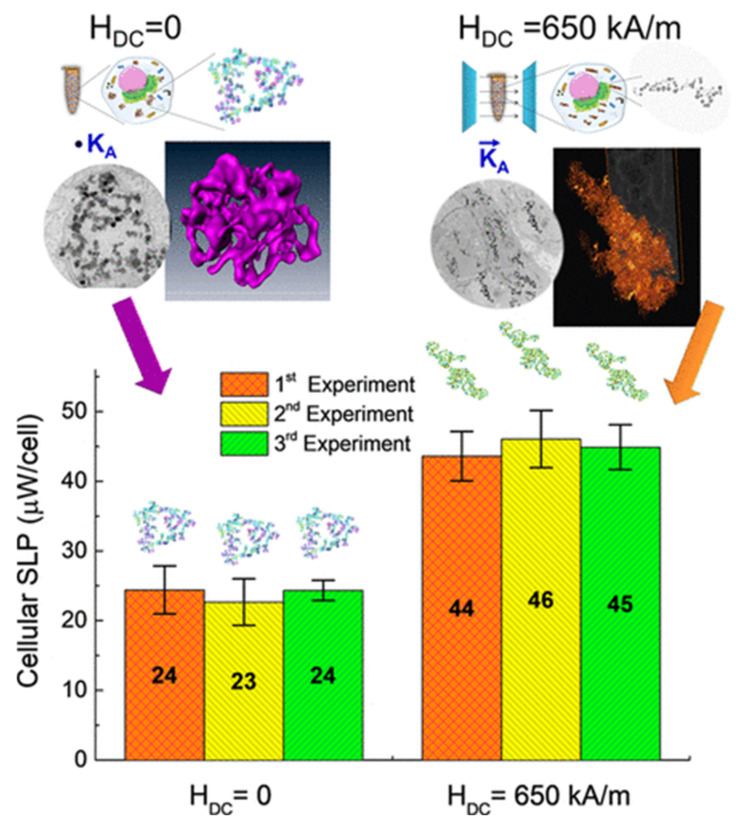
In vitro power absorption experiments for aggregates of MnFe_2_O_4_ NPs within BV2 cells. The aggregates formed overnight without an applied field, resulting in sphere-like shape, and under an applied dc field H = 650 kA/m, resulting in elongated shape. SLP values are given per cell in μW/cell. Adapted with permission from Ref. [218], Copyright 2020 American Chemical Society.

Sanz et al. tested the heating capacity of MnFe_2_O_4_ NPs (~50 nm average size) loaded in cells cultured in a static magnetic field of 650 kA/m and in no field [218]. The application of the magnetic field led to the formation of linear aggregates inside the cells, in contrast with the spherically shaped ones that formed in absence of field; in vitro measurements indicated that the heating efficiency was approximately a factor 2 higher in the first case (Figure 9).

## 6. Hybrid Systems

Hybrid magnetic materials, consisting of NPs loaded into a non-magnetic organic or inorganic matrix, are generally created to combine different functionalities coming from the magnetic NPs and the matrix. Thus, in addition to the heating capacity and the enhancement of contrast in diagnostic imaging that the NPs provide, the matrix may allow drug transportation (hydrophobic and hydrophilic) and stimuli responsive actions, eventually improving the theranostic concept [16,219,220,221,222,223,224]. If the containers in which the NPs are encapsulated are also of nanometric dimensions, they are often referred to with the terms ‘nanocontainers’, ‘nanocapsules’, ‘nanovectors’, ‘nanocarriers’. Therefore, in this formulation, the magnetic NPs are spatially confined at the nanoscale and generally subjected to interparticle dipolar magnetic interactions, whose strength varies with their concentration. Moreover, the NPs are not free to move and their spatial arrangement depends to some extent on the geometric and chemical-structural features of the carriers into which they are loaded.

The methods for the synthesis of these hybrid systems are numerous and vary mainly according to the type of matrix and the characteristics of the loaded magnetic NPs. Here below, we focus on three types of container: liposomes, polymeric matrix and silica.

From the magnetic point of view, the magnetothermal phenomenology is basically similar to that already described in Section 4.2, i.e., typical of aggregates of dipolar interacting NPs. As we have discussed, dipolar interactions between NPs of a sphere-like aggregate play a complex role in the heating mechanism, not always favorable for SAR. However, it is worth remarking that hybrid systems are intended as multifunctional agents. If the thermal degradation of the carrier and the release of drug must be induced, the amount of loaded NPs must be high enough to generate the needed heating power, even if their accumulation may determine the onset of strong dipolar interactions and lead to a decrease in their thermal response. Moreover, a high amount of magnetic NPs is needed for efficient imaging and for enhanced magnetophoretic mobility and tissue targeting.

Although this review is devoted to artificial magnetic systems, it is worth noting that also NPs internalized in cells constitutes hybrid systems. In fact, it is now quite well established that inorganic NPs taken up by cells are concentrated within intracellular vesicles having a typical size of some hundreds of nanometers (late endosomes and lysosomes). Cellular internalization leads to a considerably reduction of the NP heating efficiency. Cabrera et al. carried out an in-depth study of this phenomenon and demonstrated that the inhibition of the Brownian relaxation process, caused by the immobilization of the NPs inside the cells, accounted only in part for the heating reduction [162]. The main cause was the intracellular clustering of the NPs, which favored the dipolar interactions. Levy et al. investigated the magnetic outcome of iron oxide NPs, with size below 10 nm, injected intravenously into mice [161]. The superparamagnetic behavior of the NPs was modified following cellular uptake and confinement within intracellular vesicles, due to the dipolar interactions. A different increase in the blocking temperature T_B_ occurred depending on whether the NPs were internalized in the liver, spleen or adipose tissue, reflecting their different arrangement inside the cells. The authors used the analysis of the dynamical magnetic behavior of the NPs as a tool to gain a fundamental understanding of the local organization of the NPs in the intracellular compartments.

The influence of dipolar interactions on the magnetic heating properties of NPs in lysosomes was also theoretically studied by Tan et al. using Monte Carlo simulations [225]. A spatial repartition of the heating power inside the lysosomes was demonstrated, related to changes in the local concentration of the NPs.

### 6.1. Liposomes

Liposomes are vesicles formed by a double layer of phospholipids and are used clinically as drug-delivery vehicles. Magnetic NPs can be encapsulated either in the aqueous or in the organic part, between the lipid bilayer, forming magnetoliposomes, which have been extensively investigated as drug-delivery system for targeting tumors and their microenvironment [226]. In addition, liposomes are thermosensitive, so an AMF can stimulate the co-encapsulated internal heating source (iron oxide NPs), for the thermally triggered drug release without increasing the environmental temperature. It should be noted that only an increase of few degrees (2–4 °C) is needed at the NP surface, in close contact with the lipid, to overcome the melting temperature of the liposome, i.e., to change it from the gel to liquid state and release the content. The presence of NPs influences the phase temperature of lipids.

**Figure 10:**
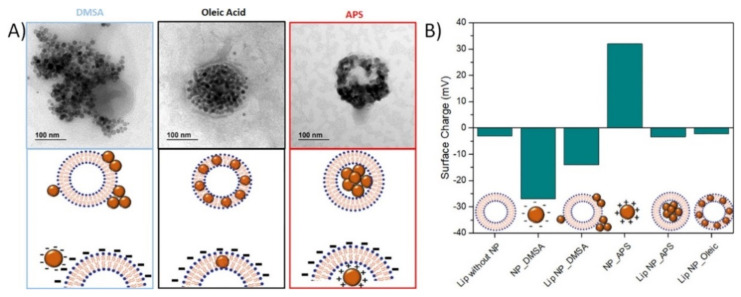
(**A**) TEM images and corresponding schemes of liposomes produced with iron oxide NPs using different coatings as dimercaptosuccinic acid (DMSA), oleic acid and (3-aminopropyl)trimethoxysilane (APS), resulting in different spatial configurations of the NPs. (**B**) Zeta potential measurements at pH 7 for these magnetoliposomes and the comparison with free NPs. Adapted with permission from Ref. [230], Copyright 2020 American Chemical Society.

The thin-film hydration method coupled with sequential extrusion is the most versatile and reproducible method for the production of magnetoliposomes. It allows incorporating the NPs, previously synthesized, in the aqueous [227] and the organic phase [228] at the same time that the liposome is formed. Different spatial distribution of the NPs can also be controlled inside or outside the liposome by changing the NP coating (Figure 10). The fact of being encapsulated inside the lipid bilayer can induce a shift of the blocking temperature T_B_ of the NPs to higher values, indicating an increased aggregation degree in comparison with free NPs. As shown by Forte Brollo et al., the aggregation degree was higher for oleic acid coated NPs, given their confinement at the lipid bilayer. Moreover, T_B_ increased following internalization of NPs into cells, namely as their intralysosomal density increased [228].

Indeed, the organic confinement can influence significantly the dynamic magnetic response of the NPs. For 9 nm and 7 nm citrated iron oxide NPs densely packed inside liposomes, under high field conditions (AMF of 27 kA/m and 700 kHz), it was observed an increase in SAR values of 438 W/g and 164 W/g compared to 270 W/g and 108 W/g for 9 nm and 7 nm NPs uniformly dispersed in colloidal suspension [219]. These data highlighted the influence of NP size and the noticeable effect of their local confinement in liposomes. The high volume fraction of NPs inside liposomes led to magnetic dipolar interactions, which could be evidenced by ZFC-FC magnetization measurements, in a shift of T_B_ to higher temperatures and a flattening of the FC curve below T_B_.

Even smaller particles (3–5 nm) encapsulated into liposomes can reach the high concentration needed (~10 mg Fe/mL) for an efficient heating (2–4 °C) under high frequency field (750–1150 kHz) [227]. If larger 16–36 nm NPs were encapsulated in liposomes, resulting in 3–6 NPs per liposome, it was observed that lipid cover did not interfere with magnetic response of the NPs (no variation in T_B_). SAR values at moderate AMF amplitude and frequency (12 kA/m and 197 kHz) were 34 W/g and 26 W/g as the particle size increased, enough to produce a temperature rise from 37 °C to 42–46 °C [229].

However, for 8 nm and 15 nm DMSA-coated NPs attached outside liposomes and under mild field conditions (<300 kHz), a slight reduction in heating efficiency for the smallest ones was reported, indicating Néel relaxation regardless being or not physically connected to the liposome; however, for 15 nm NPs, SAR dropped extensively (60%), which reflected that NPs had both Néel and Brownian relaxation [230]. Those NPs were prepared by thermal decomposition in organic media.

Finally, when 4-nm oleic-coated iron oxide NPs were embedded in the lipid membrane, a slight drop in SAR values was reported in an AMF of 20 kA/m at 500 kHz [231]; those magnetoliposomes showed a SAR value of 171.6 W/g.

### 6.2. Polymeric Matrix

Progress in modern polymer chemistry enables the design of polymeric carriers with a defined chain architectures and controlled sizes. Block copolymer vesicles, also termed polymersomes, offer an attractive structure for drug delivery applications, overcoming the limitations of instability associated with liposomes. By manipulating the co-assembly of amphiphilic co-polymers it is possible to obtain bi- or multilayer structures with an hydrophilic interior or forming stable micelles with an hydrophobic interior. Poly(ethylene oxide)–poly(propyleneoxide)–poly(ethylene oxide) triblock copolymers and poly(N-isopropylacrylamide) homopolymers have a few inherent defects such as limited biodegradability. Poly(l-lactic acid) or poly(lactide-co-glycolide) and poly(ethyleneglycol) or poly(ethylene oxide) as well as thermoreversible hydrogels like PEG-grafted chitosan/glycerophosphate and poly(organophosphazene) gels have been reported to be biodegradable.

Magnetic polymersomes having a hydrophobic internal membrane core made of the biodegradable block poly(trimethylene carbonate) and a polypeptide biocompatible corona of poly(l-glutamic acid, PGA) were loaded with hydrophobic 6.3 nm NPs, embedded into the membrane of dual-loaded vesicles by one-step nanoprecipitation [16]. The simultaneous loading of maghemite NPs and DOX was also achieved by nanoprecipitation. In an AMF of 2.12 kA/m and 500 kHz, the release of DOX was enhanced by a factor 2 compared to the same vesicles with NPs embedded in the membrane but kept away from the coil. In fact, even if the global temperature of the tested suspension remained almost unchanged, the local temperature increased by about 7 °C in the close vicinity of the polymeric membrane (i.e., at the nanometric scale), increasing dramatically the diffusion of the encapsulated DOX.

Oleic acid MnFe_2_O_4_ NPs were embedded within the amphiphilic copolymer (PBMA-g-C12) through a mini-emulsion method and showed that large NP size (here 18 nm) and a low loading ratio were preferable for a high SAR [232]. For 6 and 18 nm MnFe_2_O_4_ NPs, T_B_ decreased with the polymer immobilization and even more when the loading density was reduced. The results suggested that the polymer matrix was effective at isolating magnetic NPs, thus reducing the magnetic interactions. High SAR values of 330 W/g for 18 nm NPs (AMF of 4 kA/m and 435 kHz) were found for low polymer contents and ascribed to a relatively lower magnetic interaction due to a large amount of polymer isolating the MnFe_2_O_4_ NPs.

Other interesting biodegradable polymeric vesicles for drug delivery are those made up of PEG hydrophilic groups and polyester hydrophobic groups like PLA and PCL, forming a micelle structure with a hydrophobic interior. Sadat et al. synthesized polystyrene nanospheres with average diameter of 100 nm embedding 10 nm Fe_3_O_4_ NPs, superparamagnetic at room temperature [233]. The magnetic NPs confined in the nanospheres exhibited heating capability (in an AMF of 4.5 kA/m and 13.56 MHz, a maximum SAR ~ 70 W/g was measured), although significantly lower compared to that of free NPs of similar dimensions, mainly due to the effect of dipolar interactions. Note that due to the high field frequency used in the heating tests, Brownian relaxation could not be a dominant heating mechanism in either of the two NP systems.

Hexagon-shaped Co and Mn-doped iron oxide NPs of 14.8 nm were embedded into the hydrophobic interior of a poly(ethylene glycol)-b-poly(ε-caprolactone) (PEG–PCL)-based polymeric particle, prepared by solvent evaporation approach [234]. The developed nanoclusters exhibited enhanced heating efficiency (SAR ~ 1.2 kW/g in AMF of 26.9 kA/m and 420 kHz) when compared to the individual NPs (SAR ~ 1 kW/g), indicating a favorable effect of dipolar interactions between the NPs following their confinement in the polymer. In vivo studies demonstrated that magnetic hyperthermia mediated by the nanoclusters significantly inhibited the growth of tumors.

Similar results were found for smaller NPs immobilized in a similar polymer. Oleic acid coated 8 nm NPs prepared by coprecipitation were encapsulated in a biodegradable polymer poly-ε-caprolactone (PCL) employing the oil-in-water emulsion/solvent evaporation method [235]. As the NP size distribution and anisotropy were reasonably assumed not to vary during the encapsulation into the polymeric capsules, the observed increase in T_B_ was exclusively ascribed to changes in the dipolar magnetic interactions caused by the particle aggregation state. SAR increased with the loading into the polymer (AMF of 15.9 kA/m and 337−869 kHz). Additionally, the hypothesized ligand exchange with the carboxylic groups of the PCL shell and the higher viscosity of the polymer, compared to water, were proposed to modify the Brownian relaxation time by friction of the surface of the NPs with the carrier matrix, affecting the overall heating transfer mechanism.

Some studies reported about hybrid systems made of magnetic NPs loaded in nanocapsules of poly(lactic-co-glycolic acid) (PLGA)—a copolymer approved for human use and largely employed for drug-delivery applications due to its good biodegradability and biocompatibility—with the aim of realizing highly biocompatible nanovectors, which, by virtue of their magnetic functionality, can be possibly driven to a specific target, where they can release their load, eventually exploiting the heating capacity to promote the degradation of the PLGA matrix [236,237,238,239,240].

**Figure 11:**
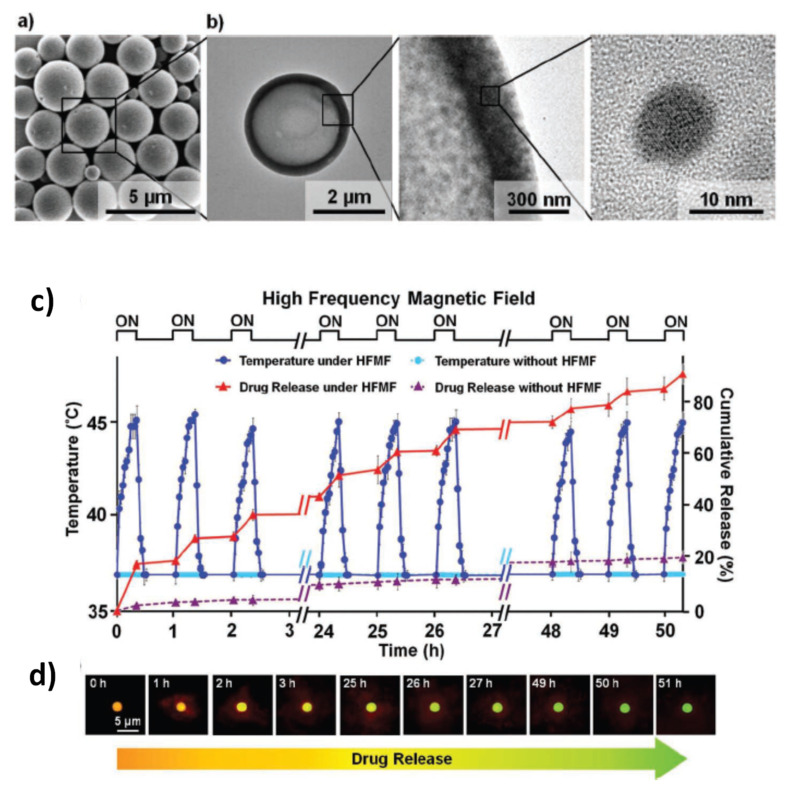
(**a**) SEM image of the PLGA hollow microspheres. (**b**) TEM images revealing the hollow structure of one microsphere and the single crystalline structure of the encapsulated iron oxide NPs. (**c**) Release profiles of DOX from the PLGA hollow microspheres, incubated in PBS, under AMF (2.5 kA/m and 50–100 kHz); the AMF was switched between “ON” and “OFF” modes. Their counterparts, not stimulated by AMF, were used as a control. (**d**) Fluorescence micrographs showing the changes in color of the hollow microspheres during the DOX release. Adapted with permission from Ref. [238], Copyright 2012 WILEY-VCH Verlag GmbH & Co. KGaA, Weinheim.

Chiang et al. fabricated magnetically responsive hollow microspheres (1–3 µm) from PLGA using a double-emulsion method, with the polymer shell (thickness ~ 250 nm) being doped with both DiO green fluorescent and iron oxide NPs (10–12 nm in size) and the aqueous core containing DOX [238] (Figure 11a,b). Upon exposure to an AMF (2.5 kA/m and 50–100 kHz), the NPs encapsulated in the shell rapidly increased the local temperature above the glass transition temperature of PLGA, so that the PLGA chains became much more mobile. This led to an increase of the permeability of the PLGA shell and thus to a rapid release of DOX molecules. The process was reversible, namely without exposure to AMF, the local temperature relaxed back to 37 °C swiftly and the drug release terminated (Figure 11c,d).

**Figure 12:**
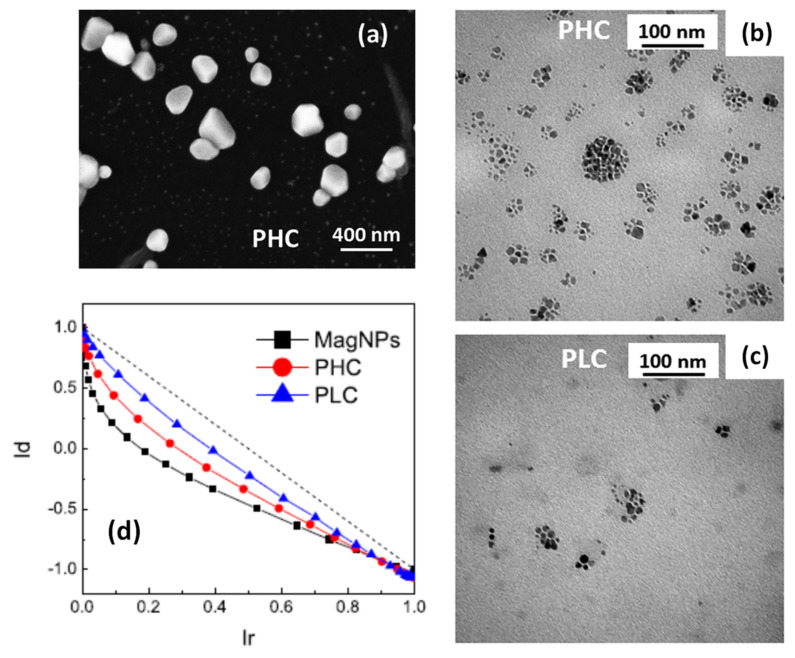
(**a**) SEM image of PLGA nanocapsules loaded with Mn-doped magnetite NPs. (**b**,**c**) Bright-field TEM images of the PLGA nanocapsules with higher (sample PHC) and lower (sample PLC) load of magnetic NPs, which provided the dark contrast. (**d**) Henkel plots for samples PHC, PLC and for the free magnetic NPs (sample MagNPs), obtained by combining the DCD and IRM magnetic remanence, measured by SQUID at T = 5 K. In particular, the normalized DCD values (Id) are plotted as a function of the normalized IRM values (Ir); the observed deviation from the linear trend (dotted line) is a measure of the strength of the dipolar interactions in the samples. Adapted with permission from Ref. [240], Copyright 2019 American Chemical Society.

Del Bianco et al. studied the structural and magnetic properties of PLGA nanocapsules (typical dimension around 200 nm) with a different load of 8 nm oleate-coated Mn-doped magnetite NPs (~5.3 and ~0.72 wt.%), prepared by oil-in-water (o/w) emulsion solvent extraction method [240]. The NPs were not homogeneously distributed into the nanocapsules and tended to form aggregates, which were larger in the case of greater magnetic load (Figure 12a–c). The system was particularly suitable for investigating the effects of the interparticle dipolar interactions on the magneto-heating properties. In fact, the experimental magnetic analyses—which also included the estimation of dipolar interaction strength by the IRM-DCD remanence method (Figure 12d)—well highlighted the effect of stabilization of the NP moments against superparamagnetism, as well as the appearance of low-remanence magnetic configurations of the NP aggregates. Higher heating efficiency was measured in the sample with the lower concentration of magnetic NPs (SAR ~ 68 W/g under an AMF of 18 kA/m and 245 kHz).

Finally, magnetic hydrogel nanocomposites are worth mentioning. Hydrogels are crosslinked polymer networks capable of absorbing large amounts of water or biological fluids. Composite materials made of temperature-sensitive hydrogel and magnetic NPs can be used as externally-actuated drug delivery systems. In fact, heat generation by the NPs under an AMF rises the temperature of the hydrogel above a critical temperature at which the hydrogel collapses, causing the expulsion of the imbibed water and of the loaded drug [241,242,243,244,245].

When using citric acid coated iron oxide NPs and copolymer comprising acrylic acid and 2-methacryloylethyl acrylate units as the backbone and poly-(ethylene glycol) and poly(N-isopropylacrylamide) as the grafts, a high amount of NPs (8–10 nm) were incorporated within the thin walls of hollow nanogels (hydrodynamic size DLS ~ 200 nm) [221].

In order to prolong the retention time of magnetic NPs localized within tumor targets and favor repeatable magnetic hyperthermia treatments, poly(organophosphazene) (PPZ) hydrogels were used to encapsulate 9 nm Zn_0.47_Mn_0.53_Fe_2_O_4_ NPs, with good heating efficiency (SLP of 150–200 W/g in an AMF of 19.5 kA/m and 389 kHz), synthesized by thermal decomposition. More than three weeks retention of NPs within tumors after a single injection of hydrogel nanocapsules (mean diameter ~ 150 nm) facilitated successful multiple magnetic hyperthermia and four cycles of treatments showed excellent anti-tumor effects on a mouse xenograft model [246].

### 6.3. Silica

Solid matrices made of inorganic materials, such as silica, are also interesting vehicles for drug delivery. They provide a biocompatible substrate with a well-controlled size dispersion and a polyvalent surface chemistry for functionalization and/or vectorization [247,248]. In contrast to most polymeric matrices, the inorganic matrices are able to protect the cargo to environmental pH or extreme biological conditions. Besides, they can be prepared with an engineered internal structure (mesoporous matrices) that maximizes the cargo capacity and may provide sophisticated methods of dose regulation [249,250,251]. Magnetic NPs are frequently inserted in this sort of matrices to provide additional functionalities, such as magnetic separation or contactless triggering that can be externally activated even in deep tissues thanks to the deep penetration of magnetic fields [252].

The most common synthesis route for the production of inorganic matrices of silica is the sol–gel condensation of silica precursors (typicaly tetraethylorthosilicate, TEOS) known as Stöber method [253]. This method consists on the hydrolisis of precursor radicals and condensation of O-Si-O groups. In the presence of the proper surfactants (typically CTAB), it is possible to create an internal structure of ordered porous with tunable sizes and shapes [248]. There are three general methods to functionalize this kind of nanostructures with magnetic NPs: (i) encapsulating them on the silica matrix [254], (ii) inserting them in the internal structure of the matrices [255] or (iii) anchoring on their surface [256].

The arrangement of magnetic NPs inside the inorganic matrices results of paramount importance for the thermal activation of the release mechanisms and the magnetic response of the overall nanostructure. The mechanism of immobilization determines how the magnetic NPs will modify their magnetic response after the integration on the silica structure. The encapsulation of NPs inside the silica matrix is generally performed by entrapping few iron oxide nanocrystals in close proximity. Depending on the number of crystals trapped inside the silica matrix, the dipolar interactions may result more or less intense, but generally the presence of a silica shell around the NPs separate the magnetic cores and reduce the effects of dipolar interactions [257].

Inserting NPs in the internal structure of the mesoporous silica is complicated since the pore size are generally ranged between 2–10 nm [258]. Thus, the size of NPs that can be fitted inside the pores is highly restricted and the strategy usually chosen to create these systems is the in situ growth of the magnetic NPs [255]. Virumbrales et al. compared the magnetic response of Zn and Ni ferrite NPs grown inside a mesoporous matrix of 2.6 nm in porous diameter [259] to similar magnetic NPs grown on amorphous matrices and with no matrix. They observed lower M_S_, higher H_K_ and higher T_B_. The authors attributed these differences to the reduced dipolar interactions between magnetic cores grown on the porous and to the effect of the matrix on their surface anisotropy. Nevertheless, it is worth noting that the superparamagnetic response of such small cores was weak and difficult to tune.

The third strategy mentioned, in which the magnetic NPs are attached on the surface of silica particles, is one of the most interesting for magnetic harvesting and for the regulation of thermosensitive coatings. In this arrangement, the silica acts as a solid spacer between magnetic NPs decreasing the possible interaction. In this case, the magnetic response can be considered similar to individual NPs, although some authors have observed spin-glass behavior at very low temperatures for silica particles densely coated with iron oxide NPs [260].

## 7. Mixed NP Systems

Recently, some articles have reported on a promising new strategy that could be undertaken to regulate the heating capacity of magnetic NPs. It basically consists in mixing together NPs with different compositional and/or structural properties and therefore with different magnetic behavior, so as to exploit the synergy arising from their combination.

A first example of this new route is represented by the article by Vamvakidis et al., who, using a microemulsion-based method, were able to incorporate soft (MnFe_2_O_4_) and hard (CoFe_2_O_4_) NPs, at the same weight concentration, into spherical and compact nanoaggregates covered by biocompatible sodium dodecyl sulfate (SSD) polymer molecules [261]. Emulsion droplets are convenient templates to confine NPs into clusters by evaporating the dispersion solvent and the diameter of the colloidal clusters can be controlled by adjustment of the emulsification process. The primary NPs had a similar mean size of ~9 nm, whereas that of the final structures was of ~81 nm. At T = 300 K and under a maximum applied field of 10 kOe, the CoFe_2_O_4_ NPs showed higher magnetization (85 emu/g) and coercivity (250 Oe) than the MnFe_2_O_4_ NPs (66 emu/g, 150 Oe) and both types were coated by oleylamine, which hindered the intimate physical contact among them within each aggregate, thus ruling out exchange interactions. The authors compared the magnetization and coercivity values of the two types of NPs to those of the bi-phasic aggregates and also to those of single-phase MnFe_2_O_4_ and CoFe_2_O_4_ aggregates. Magnetic relaxation effects were not investigated. In our opinion, this study could have helped to better elucidate some puzzling results, such as the fact that the soft MnFe_2_O_4_ did not exhibit superparamagnetic relaxation at room temperature or that the coercivity of the CoFe_2_O_4_ aggregates was ~460 Oe, i.e., considerably higher than in the un-aggregated NPs. However, the authors reached the conclusion that the overall magnetic behavior of the bi-phasic aggregates, featuring magnetization ~90 emu/g and coercivity ~250 Oe at T = 300 K, was the superposition of the properties of each phase, where the soft MnFe_2_O_4_ phase and the hard CoFe_2_O_4_ phase offered moderate coercivity and high magnetization, respectively. The heating properties of the samples were tested by the calorimetric method in a AMF of 25 kA/m and 765 kHz. A strong increase of the heating capacity was observed passing from the single NPs to the single-phase aggregates (SLP increased from 27 W/g to 104 W/g for MnFe_2_O_4,_ and from 40 W/g to 223 W/g for CoFe_2_O_4_). Remarkably, a SLP value as high as 525 W/g was measured in the bi-phasic aggregates, which the authors ascribed to the good synergy between the two magnetic phases.

Iglesias et al. studied a mixture of iron oxide NPs of two distinct origins: (i) inorganic, produced by a coprecipitation method and having a mean size of ~18 nm; (ii) biomimetic, i.e., covered by a protein obtained through a bacterial synthesis, with a mean size of ~35 nm [262]. The primary goal of the authors was to provide a composition that could be used as a platform for combining drug delivery and hyperthermia. In fact, the biomimetic NPs presented an isoelectric point below neutrality and could be used for drug transportation and release at the acidic tumor environment; the inorganic iron oxide NPs showed a zero-zeta potential at pH 7 and appeared to be suitable as magnetic hyperthermia agents. Heating tests carried out on the biomimetic and on the inorganic NPs in a AMF of 18 kA/m at three different frequencies (197 kHz, 236 kHz, and 280 kHz) indicated a greater heating efficiency of the latter in all conditions. However, the highest SAR values (up to 96 W/g at 280 kHz) were measured on a mixture containing [25% biomimetic + 75% inorganic] NPs. A different mixture made of [60% biomimetic + 40% inorganic] NPs showed a reduced heating capacity compared to that of inorganic NPs alone, but better than that of biomimetic NPs. The authors associated the best heating performance of the first mixture to an improved colloidal stability of the inorganic NPs, which constituted the larger fraction, thanks to the presence of the biomimetic ones and to the steric and electrostatic repulsion between them due to the protein coating. This explanation disregarded the possible role of magnetic interactions between the two types of NPs in ruling the magneto-heating properties of the mixture. Indeed, the authors reported that both the inorganic and biomimetic NPs showed superparamagnetic behavior at room temperature, being T_B_ equal to 103 K and 145 K, respectively. However, they also measured a considerably higher T_B_ (180 K) for the mixture [25% biomimetic + 75% inorganic] NPs, which, in our opinion, was a quite clear clue of the strong influence of interparticle interactions on the magnetothermal behavior of the system.

**Figure 13:**
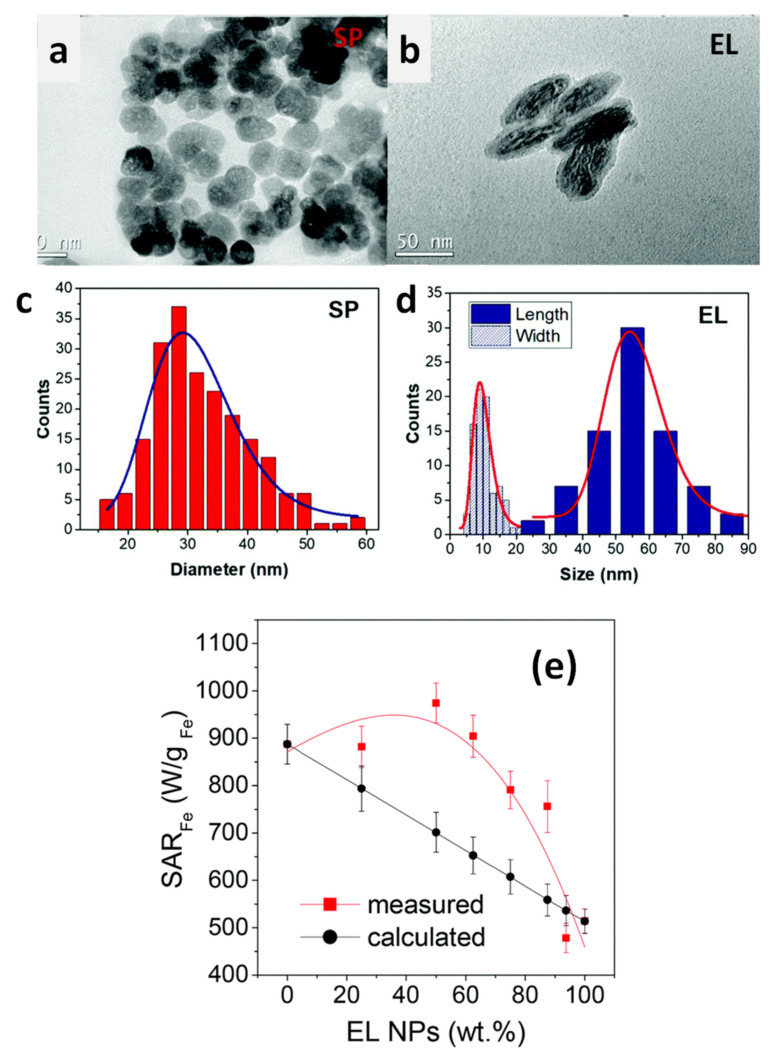
TEM images of (**a**) spherical NPs (SP) and (**b**) silica-coated elongated NPs (EL). Distributions obtained from TEM images of: (**c**) SP diameter, (**d**) length and width of the magnetic core of EL NPs. (**e**) Measured and calculated SAR values for samples obtained by mixing the SP and EL NPs in different proportions (AMF of 48 kA/m and 96 kHz). The values are shown as functions of the fraction of EL NPs in the samples. The calculated values were obtained as the weighted sum of those of the parent SP end EL NPs. Adapted from Ref. [263] under CC BY_NC 3.0 License.

Ovejero et al. studied an original magnetic system obtained by mixing together iron oxide NPs with different shape: elongated, with aspect ratio ~5.2 and mean volume of the order of 10^3^ nm^3^ (excluding the silica coating), and spherical, with mean volume one order of magnitude larger (Figure 13a–d) [263]. The first type was produced through a three-step process involving the synthesis of precursor goethite NPs, their dehydration to obtain the hematite phase (after which they were also covered with silica) and, finally, the reduction to magnetite in H_2_ atmosphere; the second type was synthesized by a polyol method. The structural features of the prepared NPs determined their intrinsic magnetic anisotropy and their magnetic relaxing behavior. In particular, the spherical NPs were essentially stable against thermal effects at room temperature (i.e., their magnetic moments were blocked), unlike the elongated ones, which, although magnetically harder for T < 100 K, became softer above 100 K and exhibited superparamagnetic relaxation at T = 300 K. The authors showed that mixing the NPs in different proportions allowed to modulate the magnetic hysteretic properties of the system, which did not correspond to the mere superposition of those of the parent NPs, but were affected by their mutual influence, described in terms of a mean field mechanism. This magnetic phenomenology directly impacted on the ability of the mixed samples to generate heat under an alternating magnetic field. In an AMF of 48 kA/m and 96 kHz, the SAR of the mixed samples varied within a wide range of values, between those of the elongated and spherical NPs (~510 W/g_Fe_ and ~890 W/g_Fe_, respectively) (Figure 13e). In general, the heating efficiency of the mixed samples was larger compared to that obtained as the weighted sum of those of the parent NPs. The highest SAR ~970 W/g_Fe_ was measured in a sample containing an equal fraction of the two types of NPs. In the author’s description, this occurred thanks to the mean field produced by the magnetically blocked spherical NPs that stabilized the thermally fluctuating moments of the elongated ones, which therefore contributed more effectively to the heat production. In short, the strategy indicated by the authors exploits one population of NPs, in the specific case the spherical ones, to potentiate the heating ability of the other population of NPs, i.e., the elongated ones. This magnetic interplay between the two types of NPs can be regulated by properly selecting their inherent structural/magnetic features and then varying the composition of the mixed samples. Thus, the creation of mixed NP systems emerges as an effective, still largely unexplored path towards on-demand adjustable magneto-heating performance.

In a completely different approach, the mixture of magnetic NP colloids with different anisotropies (composition or shape) at low concentrations has been recently proposed as a unique strategy to generate multiple local temperatures in a single reactor or biological environment [264]. At low concentrations (<0.1 mg/mL), the interactions between magnetic NPs becomes negligible and each magnetic NP can be independently activated as a local nanoheater. By tuning the field and frequency conditions of the AMF it is possible to optimize the heating performance of one or the other set of magnetic NPs (magnetite NPs with different size and shape), and thus induce an increment of the local temperature in their surrounding media. The proposed general strategy is to combine high frequency-low field vs. high field- low frequency AMF conditions in order to activate magnetic NPs with low and high anisotropy constants, respectively. An effective selective system requires a mix of at least two set of magnetic NPs with well differentiated anisotropies, what can be achieved by combining different sizes, geometries or compositions [264,265].

Such selective heating has been only applied to the activation of thermophilic enzymes that generate thermolabile products [266] or to multiplexing activation of thermosensitive membrane channels such as TPRV1 [267], but presents a tremendous potential for multiplexed contactless regulation of temperature dependent processes in biotechnology.

## 8. Conclusions

We have reviewed the main strategies that exploit the interplay between two or more magnetic elements (magnetic phases or primary NPs) to create nanosized systems with excellent heating performance. Here the term “excellent” does not refer exclusively to the achievement of higher and higher SAR values, but also to the possibility of tuning the thermal response of the nanoheaters according to the specific biomedical function they must fulfill. This is obtained through the control of the compositional and structural features of the system, which in turn determine the magnetic properties, including the nature and strength of magnetic interaction between the constituent elements. In principle, a huge number of different materials may be obtained by spanning all the possible combinations of chemical-physical characteristics. However, biocompatibility and safety constraints reduce the degrees of freedom. This aspect is crucial and, due to the increasing use of NPs in biomedicine, elucidating their interaction with the biological environment, both in vitro and in vivo conditions, as well as their fate in living organisms is currently a hot research topic [268,269,270,271], albeit beyond the scope of this review article. It is to be expected that the concerns about biosafety and long-term distribution of the NPs in the patient’s body will further push the search for innovative nanoheaters and lead to the creation of increasingly versatile magnetic architectures able to combine tunable heating efficiency with assessed biodegradation and clearance properties.

## Data Availability

Not applicable.
